# Discovery of Novel Sultone Fused Berberine Derivatives as Promising Tdp1 Inhibitors

**DOI:** 10.3390/molecules26071945

**Published:** 2021-03-30

**Authors:** Elizaveta D. Gladkova, Arina A. Chepanova, Ekaterina S. Ilina, Alexandra L. Zakharenko, Jóhannes Reynisson, Olga A. Luzina, Konstantin P. Volcho, Olga I. Lavrik, Nariman F. Salakhutdinov

**Affiliations:** 1 N. N. Vorozhtsov Novosibirsk Institute of Organic Chemistry, Siberian Branch of the Russian Academy of Sciences, 9, Akademika Lavrentieva Ave., 630090 Novosibirsk, Russia; liza95@nioch.nsc.ru (E.D.G.); volcho@nioch.nsc.ru (K.P.V.); 2 Department of Natural Sciences, Novosibirsk State University, Pirogova str. 1, 630090 Novosibirsk, Russia; 3 Novosibirsk Institute of Chemical Biology and Fundamental Medicine, Siberian Branch of the Russian Academy of Sciences, 8, Akademika Lavrentieva Ave., 630090 Novosibirsk, Russia; arinachepanova@mail.ru (A.A.C.); katya.plekhanova@gmail.com (E.S.I.); sashaz@niboch.nsc.ru (A.L.Z.); lavrik@niboch.nsc.ru (O.I.L.); 4 School of Pharmacy and Bioengineering, Keele University, Hornbeam Building, Staffordshire ST5 5BG, UK; j.reynisson@keele.ac.uk

**Keywords:** berberine, berberrubine, cancer, Tdp1 inhibitor, DNA repair enzyme, SAR, molecular modeling, sultone, sulfonate

## Abstract

A new type of berberine derivatives was obtained by the reaction of berberrubine with aliphatic sulfonyl chlorides. The new polycyclic compounds have a sultone ring condensed to *C* and *D* rings of a protoberberine core. The reaction conditions were developed to facilitate the formation of sultones with high yields without by-product formation. Thus, it was shown that the order of addition of reagents affects the composition of the reaction products: when sulfochlorides are added to berberrubine, their corresponding 9-*O*-sulfonates are predominantly formed; when berberrubine is added to pre-generated sulfenes, sultones are the only products. The reaction was shown to proceed stereo-selectively and the cycle configuration was confirmed by 2D NMR spectroscopy. The inhibitory activity of the synthesized sultones and their 12-brominated analogs against the DNA-repair enzyme tyrosyl-DNA phosphodiesterase 1 (Tdp1), an important target for a potential antitumor therapy, was studied. All derivatives were active in the micromolar and submicromolar range, in contrast to the acyclic analogs and 9-*O*-sulfonates, which were inactive. The significance of the sultone cycle and bromine substituent in binding with the enzyme was confirmed using molecular modeling. The active inhibitors are mostly non-toxic to the HeLa cancer cell line, and several ligands show synergy with topotecan, a topoisomerase 1 poison in clinical use. Thus, novel berberine derivatives can be considered as candidates for adjuvant therapy against cancer.

## 1. Introduction

**Figure 1:**
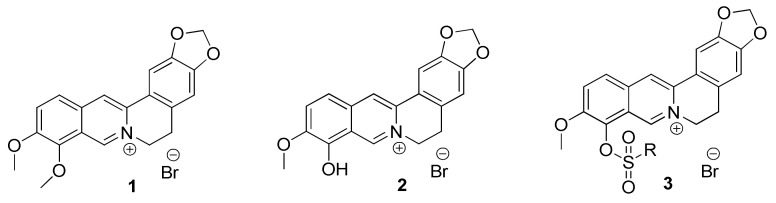
The molecular structures of isoquinoline alkaloid berberine **1**, berberrubine **2** and sulfonate derivatives of 9-demetoxyberberine **3**.

The isoquinoline alkaloid berberine **1** (Figure 1) is one of the most widespread members of the protoberberine alkaloids family, and is found in plants of the Rhoeadales, Ranunculáceae, Berberidaceae, Menispermáceae, Rutaceae and other families [1]. Berberine is primarily known for its hypoglycemic and hypocholesterolemic activity [2,3,4], and other indications are also known [5,6].

A common method to obtain improved biological activity involves chemical modifications of a hit or lead compound.

The addition of various functional groups on the berberine scaffold is widely used to create highly active agents with antibacterial, fungicidal, hypocholesterolemic, and anticancer effects [7,8,9,10]. The sulfonate moiety, which is common in, for example, cardiovascular and antiviral drugs, was used as pharmacophore to the enhance hypolipidemic properties of berberine derivatives. The reaction of the demethylated analogue of berberine, berberrubine **2** (Figure 1), with sulfochlorides was previously used to obtain 9-*O*-sulfonates of berberine **3** (Figure 1), described as hypocholesterolemic [11] and anti-inflammatory [12] agents. We have shown that berberine aryl-9-*O*-sulfonates **3** inhibit DNA-repair enzyme tyrosyl-DNA phosphodiesterase 1 (Tdp1).

**Figure 2:**
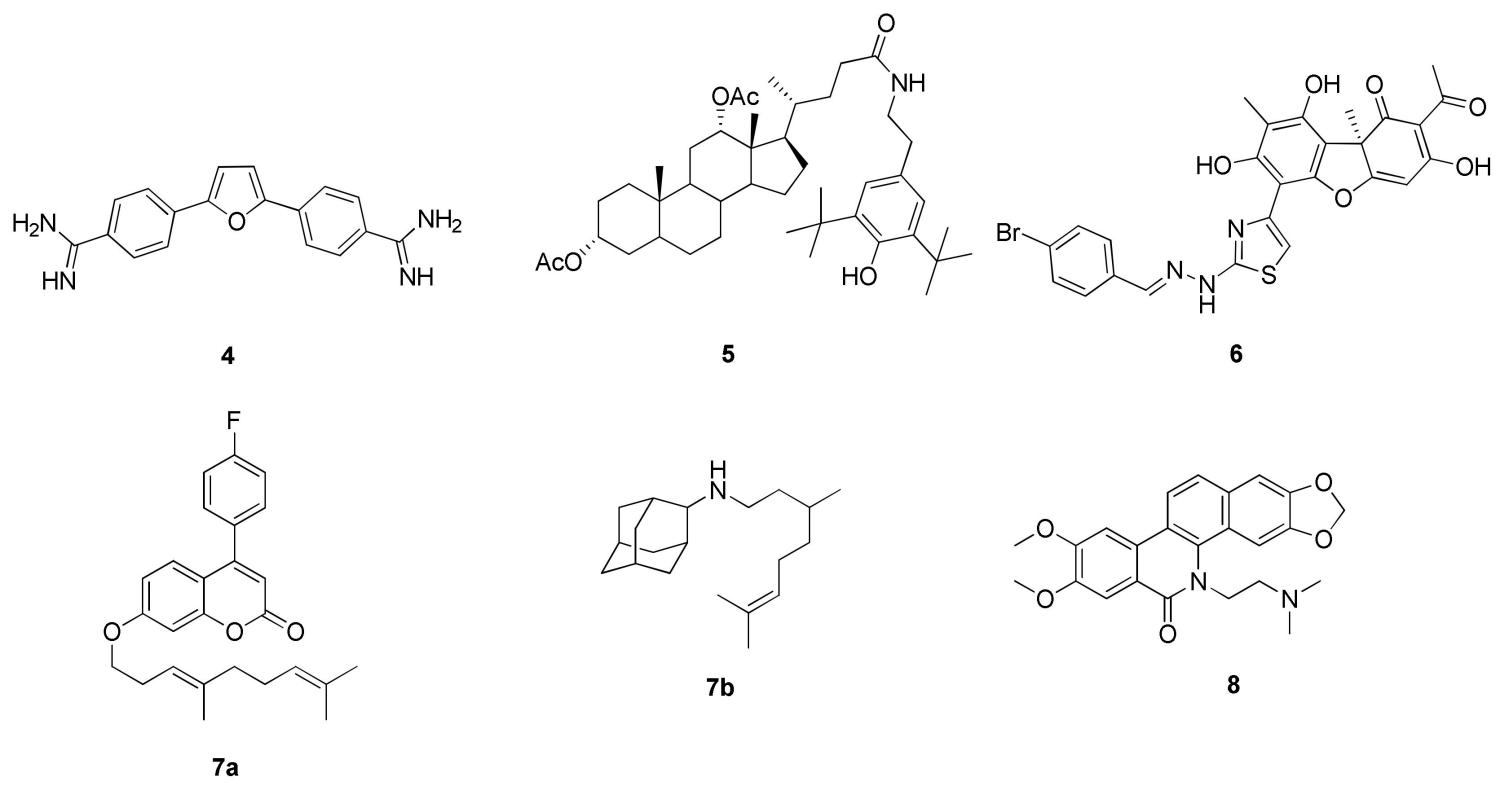
A few classes of known Tdp1 inhibitors.

Tdp1 is a eukaryotic enzyme that removes the 3′ ends of DNA after aberrant topoisomerase activity, and can process blocked 3′ ends generated by DNA damaging agents and nucleoside analogs in addition to hydrolyzing 3′-phosphotyrosyl residues (reviewed in Reference [13]). The hypothesis that Tdp1 is responsible for drug resistance in some cancers is supported by a number of studies: Tdp1 deficiency in Tdp1 knockout mice and in human cell lines with a mutation, which reduces the activity of this enzyme, leads to hypersensitivity to camptothecin or its derivatives [14,15,16]. Suppression of Tdp1 expression with minocycline enhances the antimetastatic effect of irinotecan and increases the lifespan of the experimental animals [17]. Conversely, in cells with increased Tdp1 expression, Top1 poisons cause less DNA damage [18,19]. Furthermore, Tdp1 overexpression protects colorectal cancer cells from irinotecan mediated cell death [20]. Thus, Tdp1 is a promising therapeutic target, and its inhibitors are expected to significantly synergize the effects of current anti-tumor therapies, including topoisomerase poisons and other DNA damaging agents. Indeed, it was found that combined treatment of tumor cells with Tdp1 inhibitors and anticancer drugs camptothecin or topotecan greatly increased the activity of these pharmaceuticals in in vitro [21,22,23,24,25,26,27,28] and in vivo [29,30] experiments. Nevertheless, to the best of our knowledge, no Tdp1 inhibitors have reached human clinical trials. A few classes of Tdp1 inhibitors, including natural products derivatives, are known such as furamidines **4** [31,32], derivatives of bile acids **5** [33], of lichen metabolite usnic acid **6** [34], monoterpenoid derivatives **7** [22,35] and oxinitidine **8** [36] with inhibitory activity in the micro or submicromolar ranges (Figure 2).

Berberine is known to enhance cancer cell chemosensitivity to irinotecan, semisynthetic derivative of camptothecin. Additionally, the berberine anticancer effect could be enhanced via derivatization [37]. Berberine aryl-9-*O*-sulfonates **3** as Tdp1 inhibitors sensitize HeLa cells to the anticancer drug topotecan [38]. Such a mechanism of action suggests an enhancement of the effect of established chemotherapeutic drugs, making inhibitors of Tdp1 a promising new adjunctive anticancer therapy.

According to the results of the above-mentioned work by Gladkova et al. [38], it should be noted that berberrubine in the reaction with aliphatic sulfochlorides gives the corresponding 9-*O*-sulfonates in low to moderate yields, without any information about other reaction products.

The crystal structure of Tdp1 has been available for molecular modelling studies to support the development of new inhibitors, e.g., for the binding predictions of newly synthesized derivatives [39,40], in guiding the synthetic work of new inhibitors [29,35] and finally in identifying new hit matter using structure-based virtual screening [21,33]. Recently, a crystal structure of Tdp1 was published with a co-crystalized ligand [41]; it was found by keeping three crystalline water molecules that the binding mode of the co-crystalized ligand reproduced verifying the robustness of the model [25]. It can therefore be stated that a reliable molecular model has emerged that correlates with activity results.

The primary aim of this study was to investigate the reactions of berberrubine with aliphatic sulfochlorides, identification of their main reaction pathways, and isolation and characterization of the reaction products. Furthermore, we wished to assess the inhibitory activity against the Tdp1 of the products.

## 2. Results and Discussion

### 2.1. Chemistry

#### 2.1.1. Reaction of Berberrubine **2** with Alkylsulphochlorides

The formation of sulfonates during the reaction of berberine and its derivatives with RSO_2_Cl is well-known [11,38]. We have shown earlier that the interaction of berberrubine **2** with alkyl sulfochlorides **9a**–**d** at standard conditions (methylene chloride as a solvent and triethylamine as a base) leads to the formation of sulfonates **3a**–**d** in 7–50% yields [38]. The target **3a**–**d** sulfonates were isolated by filtration from the reaction mixture. While in some cases the yield of the target sulfonates was low, the composition of the mother liquor has not been studied in any of the works mentioned.

**Scheme 1:**
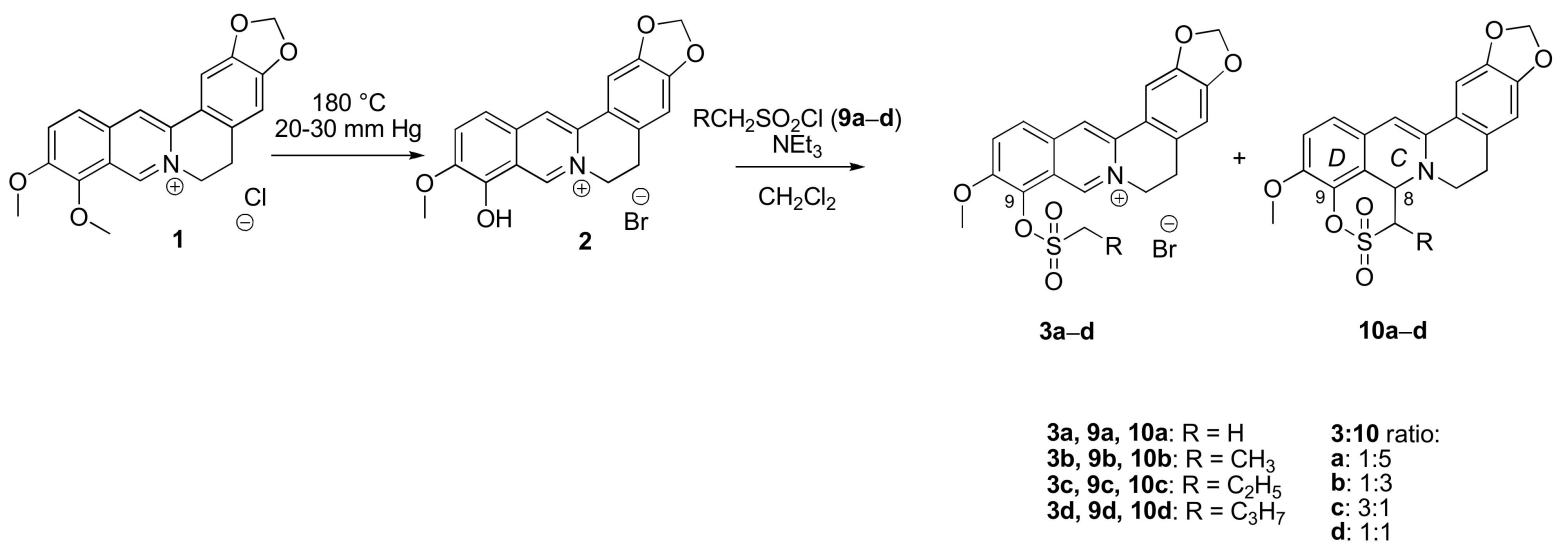
Reaction of berberrubine **2** with alkylsulphochlorides **9a**–**d**.

During additional investigations in the current work, we found, for the first time, that besides the expected sulfonates **3a**–**d**, cyclic sulfonates and sultones **10a**–**d** (Scheme 1), are formed and remained in the mother-liquor after filtration. To form **10a**–**d**, sulphochlorides **9a**–**d** need to react with the electrophilic and nucleophilic centers of berberine at positions 8 and 9, respectively, to form a new six-membered cycle condensed with *C* and *D* rings.

The ratio of products **3** and **10** in the reaction mixtures varied from 1:5, in the case of methanesulfonyl chloride **9a,** to 3:1 in the reaction with butanesulfonyl chloride **9c** according to NMR. In the series of alkylsulfonates **3a**–**d**, we observed a decrease in solubility in methylene chloride with an increasing length of the alkyl moiety, which resulted in more sulfonates with longer alkyl substituents precipitating, and therefore, in higher yields (yield increased from 7% for compound **3a** to 50% for compound **3d**). In order to isolate pure sultones **10a**–**d**, the mother liquor remaining after filtration of the precipitate was washed with water and then the product was purified by column chromatography on silica gel. New cyclic derivatives of berberine, sultones, were isolated in yields from 3% to 40%.

#### 2.1.2. Investigation into the Reaction Mechanism

**Scheme 2:**
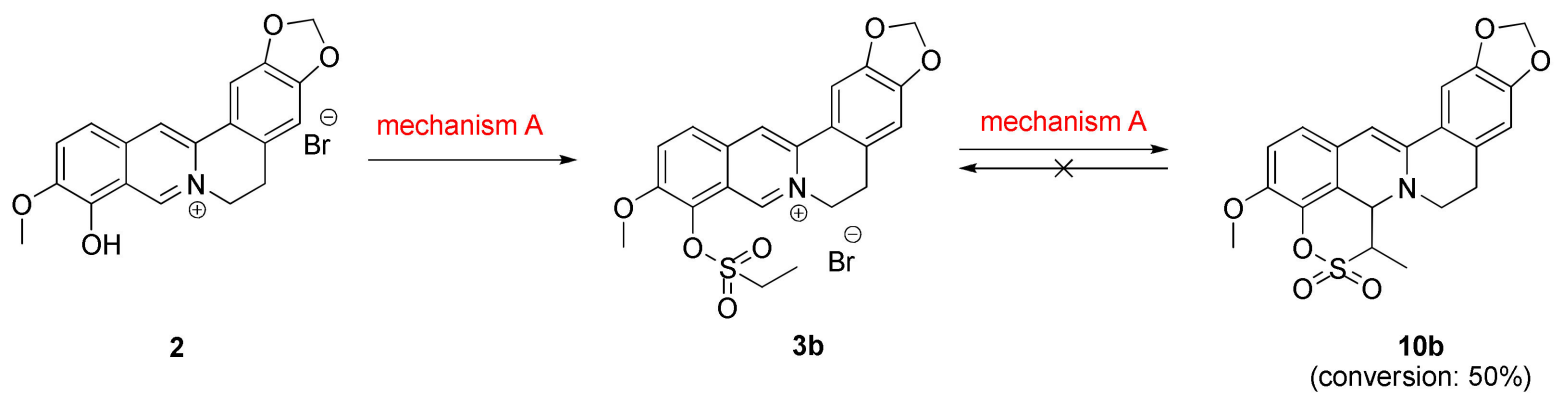
The proposed mechanism A of sultone formation.

The observed formation of cyclic and noncyclic sulfonates can be explained by a two-step reaction mechanism (mechanism A, Scheme 2) with the addition of sulfochloride at the 9-*O*-position of the berberine backbone to form sulfonate **3** and the subsequent closure of the cycle at the 8th position to form a new C-C bond leading to the formation of the sulfone **10**. To check the possibility of sequential stages processing, the sulfonate **3b** was stirred at room temperature in methylene chloride in the presence of triethylamine. In this case, a partial conversion of compound **3b** to cyclic derivative **10b** was observed (Scheme 2). However, even with prolonged stirring (for 48 h), the conversion did not exceed 50%. Nevertheless, we have shown that sultone **10b** can be formed by a stepwise mechanism. It is worth noting that incubation of individual sulfone **10b** under the reaction conditions did not lead to the formation of sulfonate **3b**, indicating the irreversibility of the second stage.

**Scheme 3:**
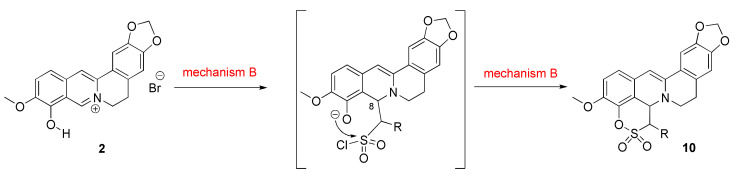
Proposed mechanism B of sultone formation.

We also cannot exclude a reaction pathway in which a nucleophilic attack at position 8 occurs initially, followed by closure of the sultone cycle (mechanism B) (Scheme 3).

**Scheme 4:**
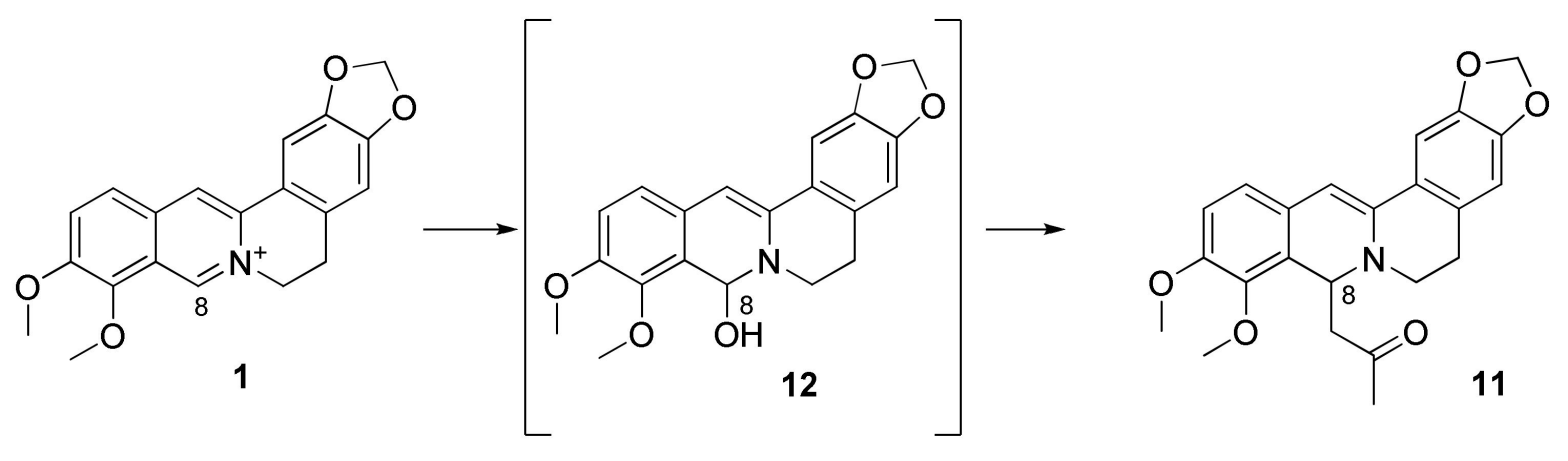
Addition of acetone molecule to berberine [42].

An example of a reaction at position 8 is the addition of an acetone molecule to berberine in alkaline conditions, leading to the formation of compound **11** [42] (Scheme 4), which, however, does not proceed directly, but through the intermediate formation of berberinol **12**. For berberrubine **2** derivatives, this type of reaction was not described in the literature.

**Scheme 5:**
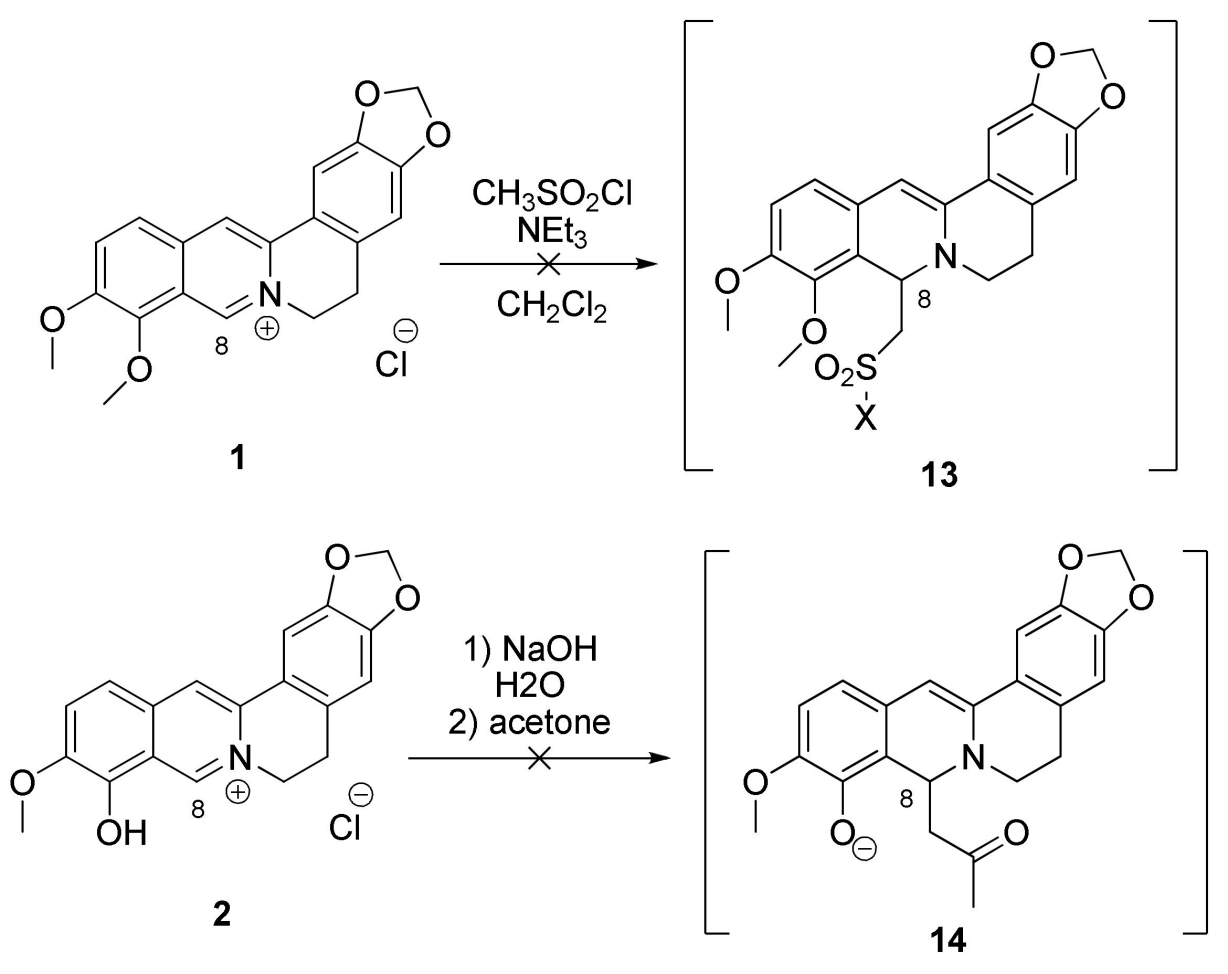
Attempts to obtain product **13** of the interaction of berberine **2** with alkylsulphocloride **9a** and berberrubinol **14.**

To investigate the possibility of primary alkylsulfochloride addition at position 8, we incubated berberine **1** under reaction conditions with sulfochlorides, including various bases (triethylamine, NaOH), but no sulfochloride addition products at position 8 (compound **13**) were observed according to NMR. In addition, we have shown that incubation of berberrubine **2** in the presence of NaOH in acetone does not lead to the formation of the 8-OH derivative **14**, the assumed intermediate in the reaction of the addition of alkyl substituents at position 8 (Scheme 5).

**Scheme 6:**
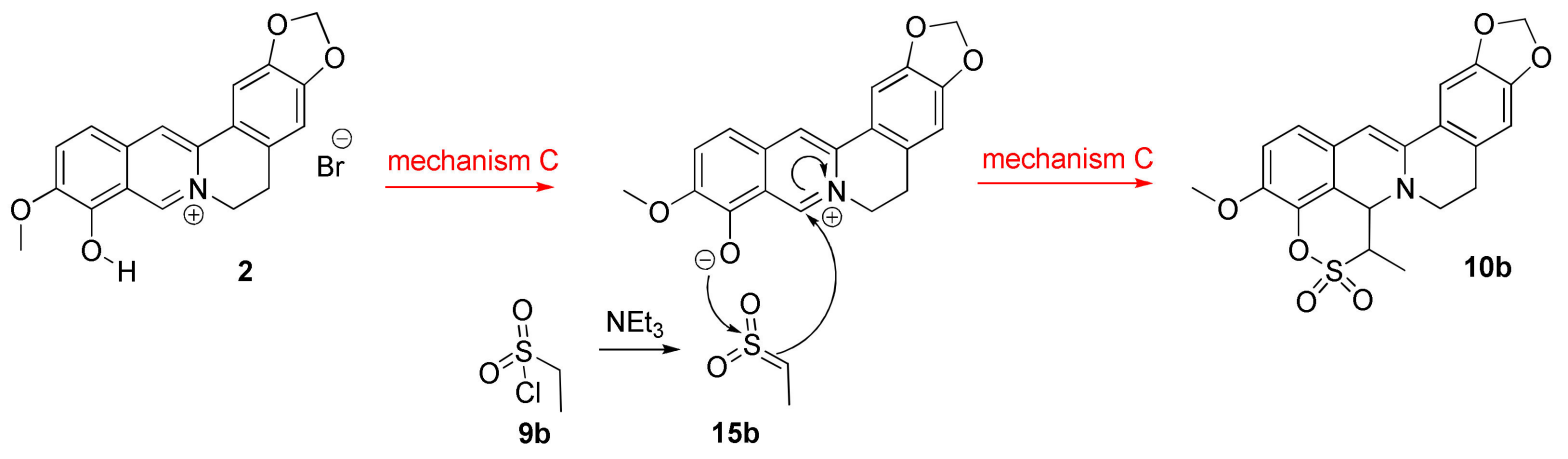
Possible mechanism C of sultone formation.

The third possible reaction pathway is a synchronous process, a one-step cycloaddition reaction (mechanism C). This path implies the formation of sulfene **15b** from sulfochloride **9b** under the action of the base. The sulfene’s dipole attacks both the electrophilic and nucleophilic centers of the berberrubine backbone (Scheme 6).

**Scheme 7:**
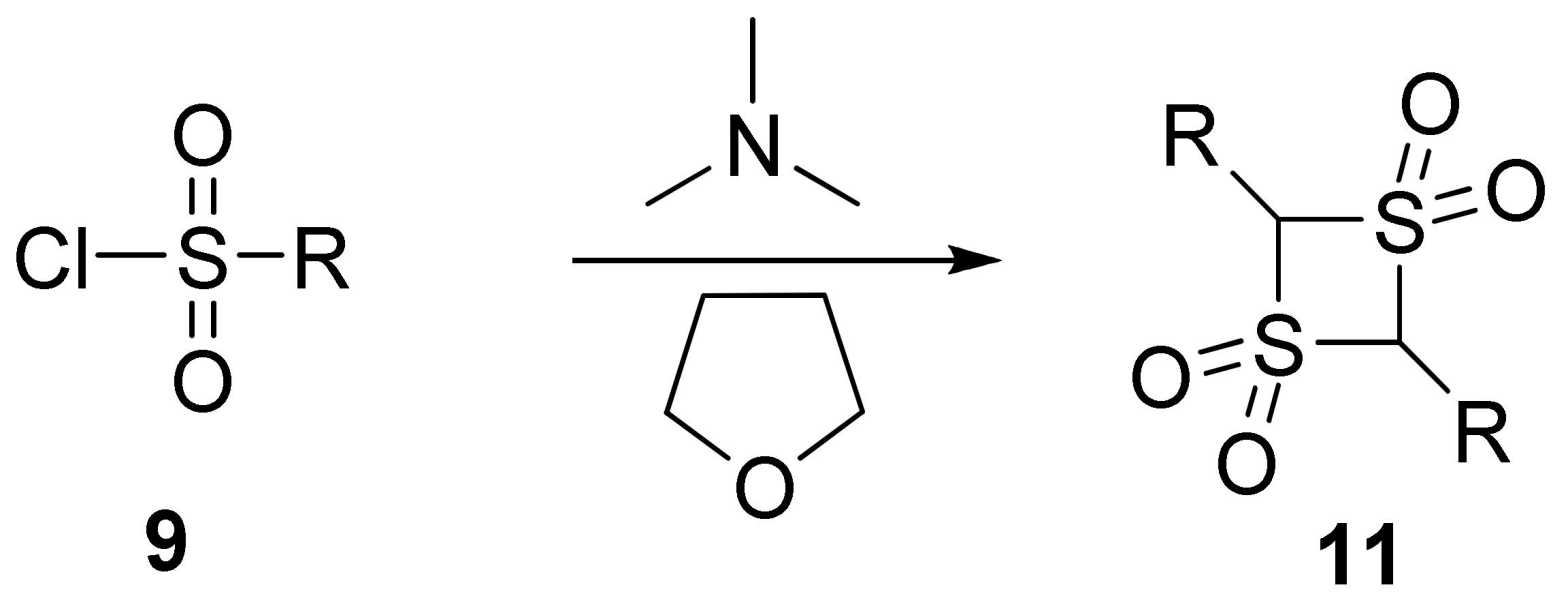
Formation of dimers from sulphenes [43].

Since the synchronous process (mechanism C) involves sulfene **15b**, and to determine the possibility of this reaction pathway, we carried out an experiment in which sulfene **15b** was initially generated from sulfochloride **9b** by adding an equimolar amount of triethylamine. When the resulting sulfene **15b** was added to berberrubine **2**, the reaction proceeded with a low conversion (~10% in 7 h). Changing the order of the reagent’s addition, by adding the berberrubine solution to the sulfene solution, led to a significant increase in the conversion of berberrubine **2**, reaching 60% in 4 h. We found that reducing the concentration of sulphochloride **9b** from 0.02 mmol/mL to 0.008 mmol/mL, and consequently the concentration of sulfene **15b**, allows the reaction to proceed with a 100% conversion in the same 4 h. It may be related to the ability of sulfenes to form dimeric structures of type **11** in concentrated solutions [43] (Scheme 7).

**Scheme 8:**
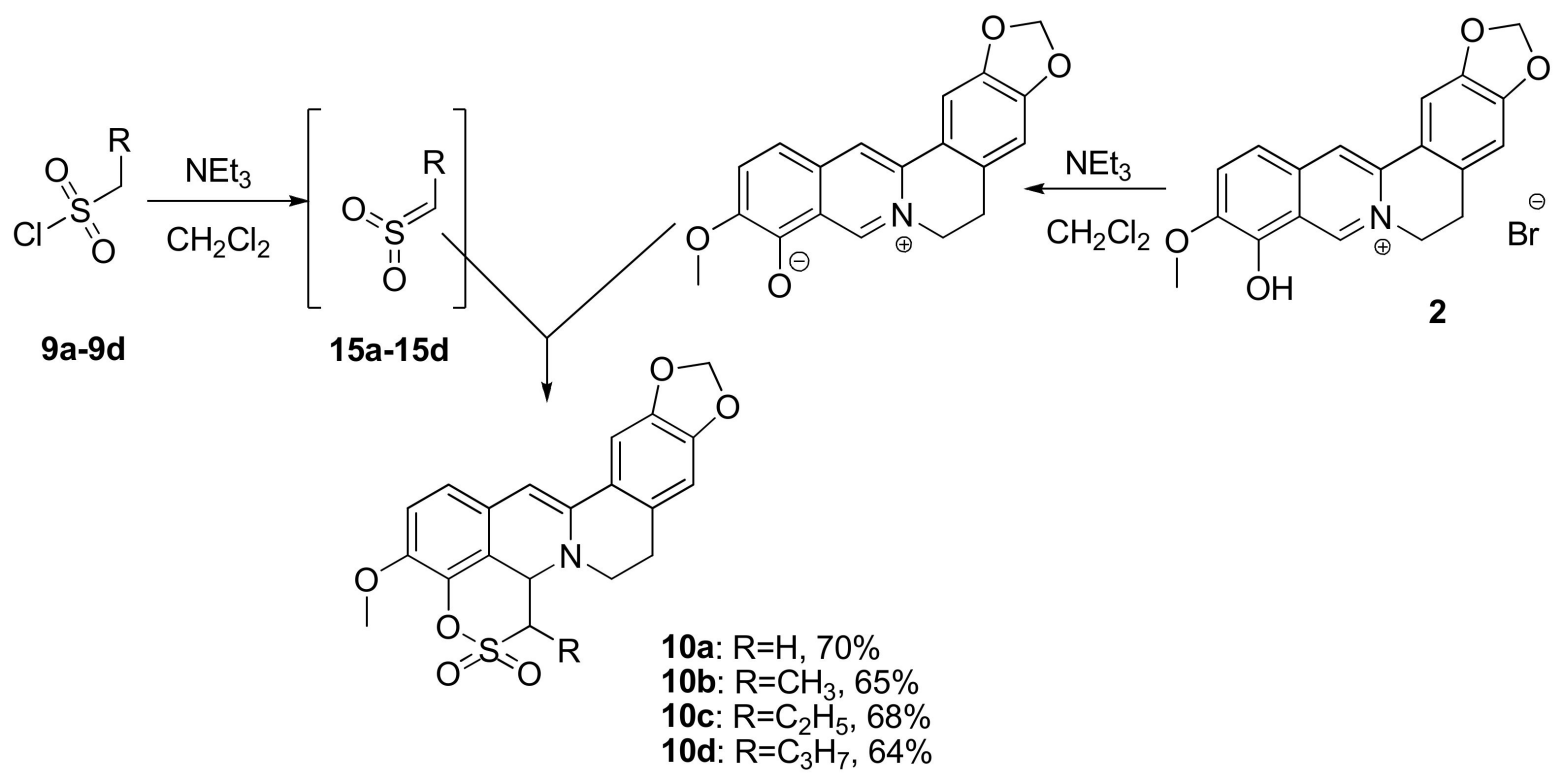
Design of the experiment leading to selective formation of sultone **10a**–**d** from berberrubine **2** and alkylsulphochlorides **9a**–**d**.

Decrease of the sulfene **15b** solution concentration and slow addition of a previously prepared mixture of berberrubine **2** and triethylamine in methylene chloride allowed us to perform the reaction with a complete conversion in 4 h and to only selectively obtain cyclic sulfonate **10b** in high yields without sulfonate **3b** impurities. This technique was extended to the reaction with other sulfochlorides, the target sulfonates **10a**–**b** were isolated in yields of 64–70% after column chromatography (Scheme 8). 

Thus, the analysis of the data obtained in the described series of experiments strongly indicates the formation of sultones **10a**–**d** by two mechanisms: sequentially, through the formation of sulfonates **3a**–**d** and the subsequent closing of the sulfone cycle leading to sultones **10a**–**d**, and by the synchronous addition of the sulfene intermediate. By changing the order of reagent addition, we can influence the reaction pathways and hence the composition of the reaction mixture. We found that, when sulfochlorides are added to berberrubine **2**, 9-*O*-sulfonates of berberrubine are predominantly formed; when berberrubine **2** is added to pre-generated sulfenes, sultones are the only isolated products.

#### 2.1.3. Structure Elucidation

**Figure 3:**
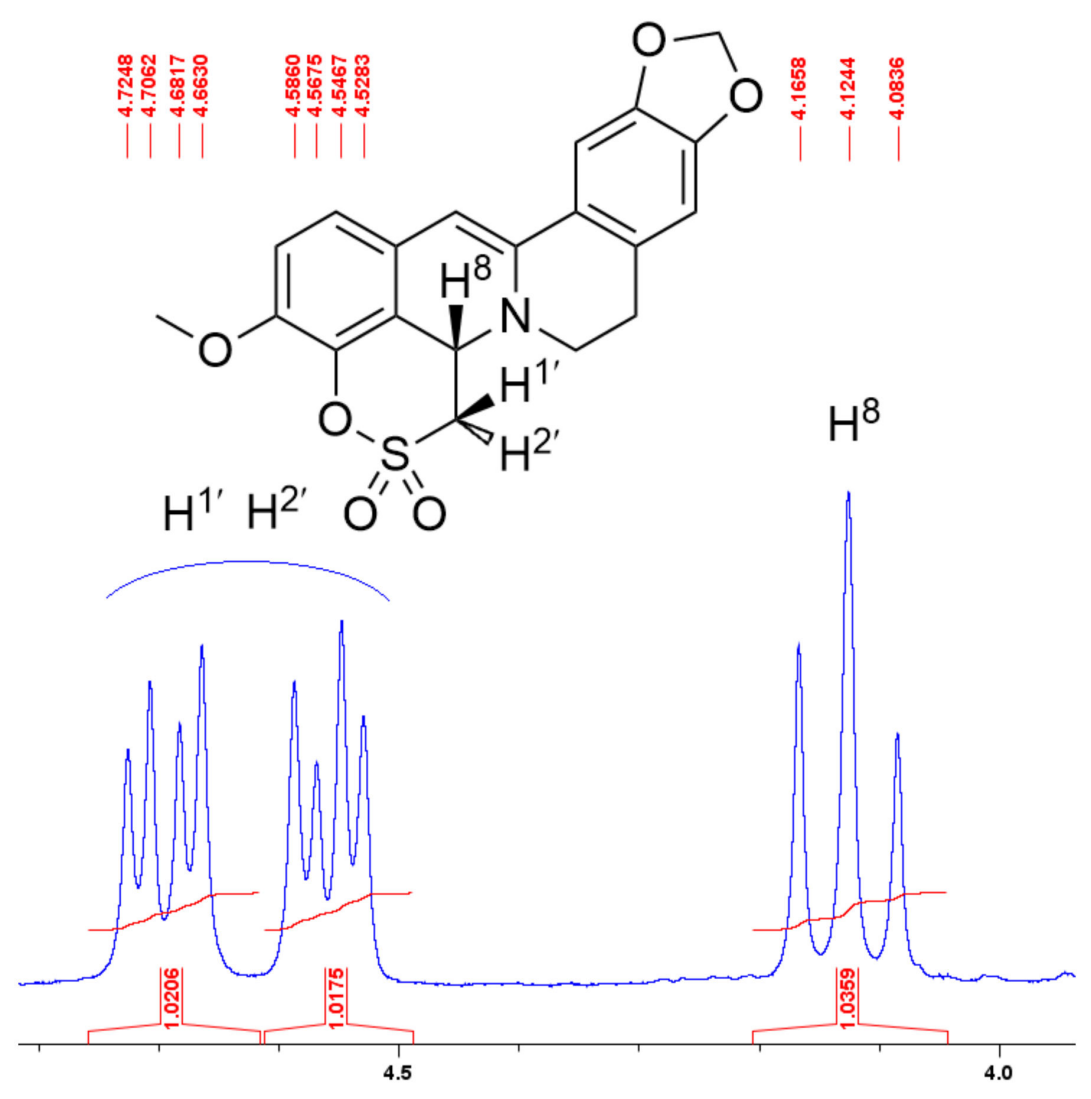
Fragment of ^1^H NMR spectrum of compound **10a**.

The molecular structures of the sultones **10a**–**d** were determined using NMR and HR-MS. In the ^1^H NMR spectrum of compound **10a**, the characteristic signals of the sultone cycle are observed (Figure 3). The protons H-8, H-1′ and H-2′ represent the AB_2_ system. The signals of H-1′ and H-2′ protons have the same multiplet (doublet of doublets). The signal of the H-8 proton has the form of a triplet.

**Figure 4:**
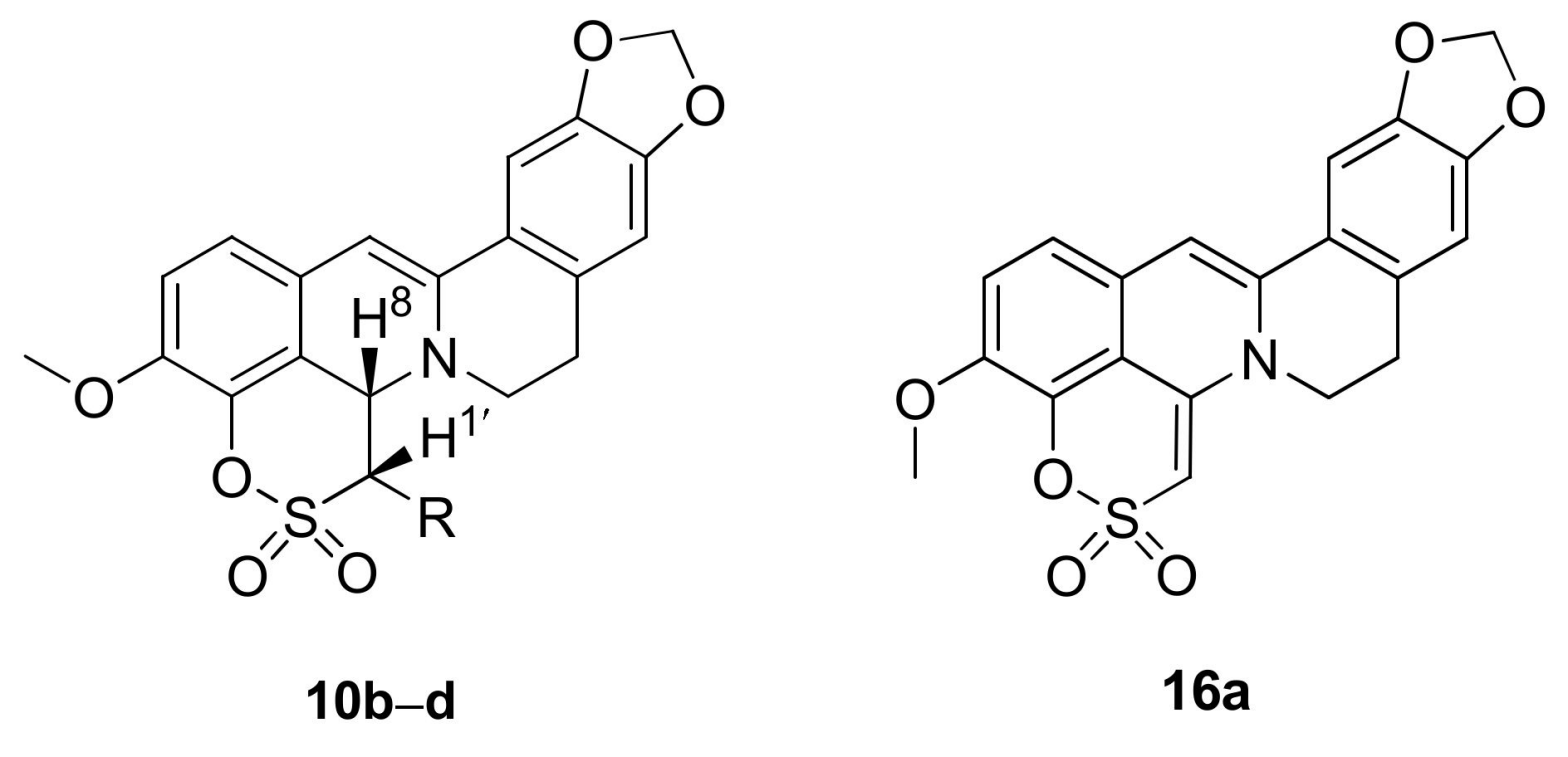
Structures of sultones **10b**–**d** and dehidrosultone **16a**.

In the ^1^H NMR spectra of the cyclic sulfonates **10a**–**d,** one set of signals is present. The H-8 proton signal in the spectra of compounds **10b**–**d** is in the form of a doublet. The spin–spin interaction constants of H-8 and H-1′ protons vary from 4.81 to 5.07 Hz, suggesting the formation of isomers with cis-location of these protons (Figure 4). The molecular weights of the sultons **10a**–**d** correspond to the calculated values.

The formation of a new C-C bond was also confirmed by 2D NMR. In the COSY spectrum of compound **10b**, the characteristic cross peak of H-8 and H-1′ protons is present.

**Figure 5:**
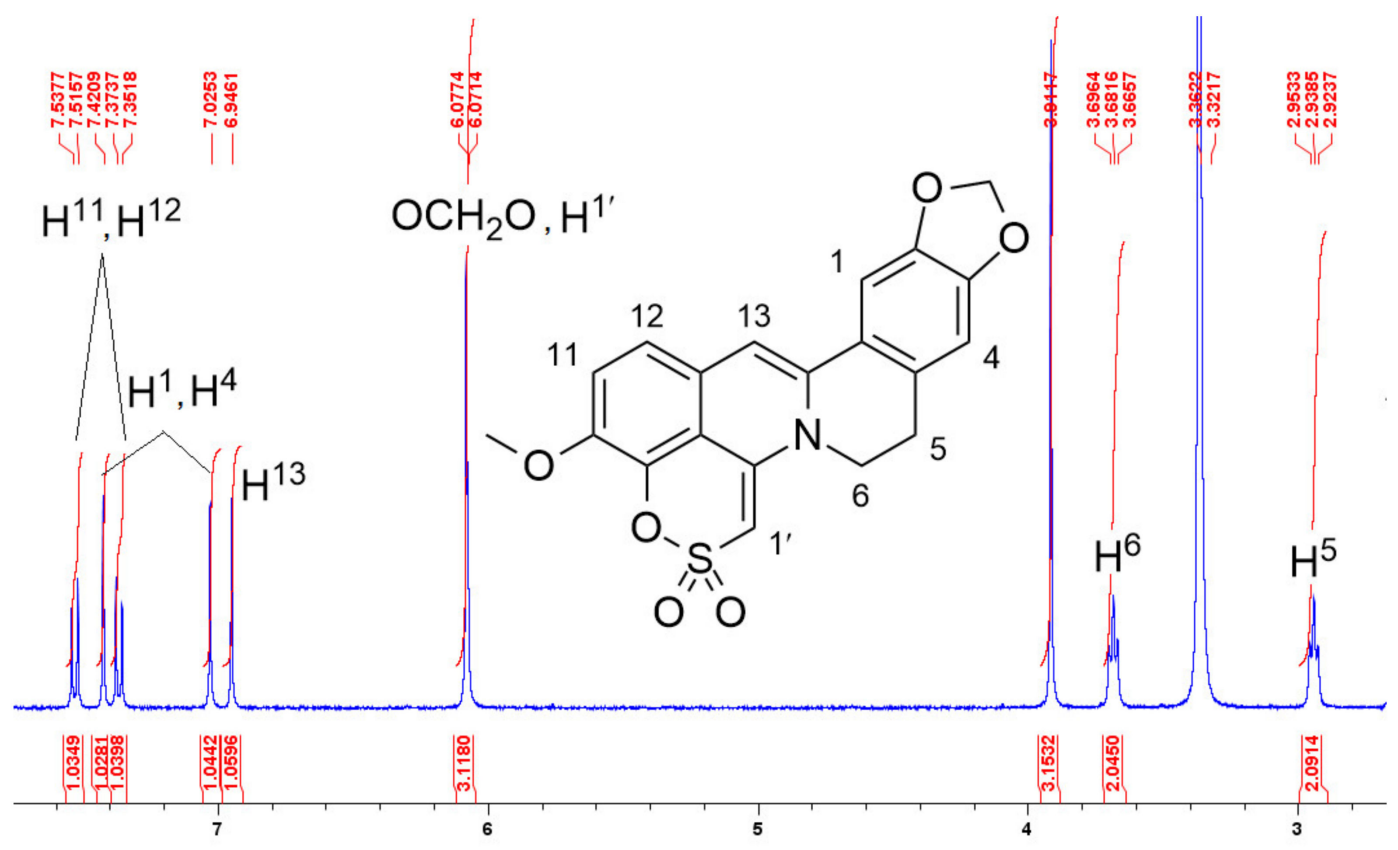
^1^H NMR spectrum of compound **16a**.

We have shown that, upon incubation in the NMR tube in DMSO-d_6_, the cyclic sulfonate **10a** partially transforms into compound **16a** (Figure 4). Dehydrosulphonate **16a** was isolated in small amounts by column chromatography and its structure was suggested based on ^1^H NMR and HR-MS spectra. In the ^1^H NMR spectrum of compound **16a**, the disappearance of H-1′, H-2′ and H-8 protons signals was observed (Figure 5), but a new signal in the near-aromatic region (δ = 6.07 m.p.) relating to the H-1′ proton appeared. This strong shift into the weak field is due to the formed double bond, which is conjugated to the aromatic system of the berberine scaffold. In the mass spectrum of compound **16a,** a peak of 397.0618 is observed, which corresponds to the product of dehydrogenation of compound **10a.**

**Scheme 9:**
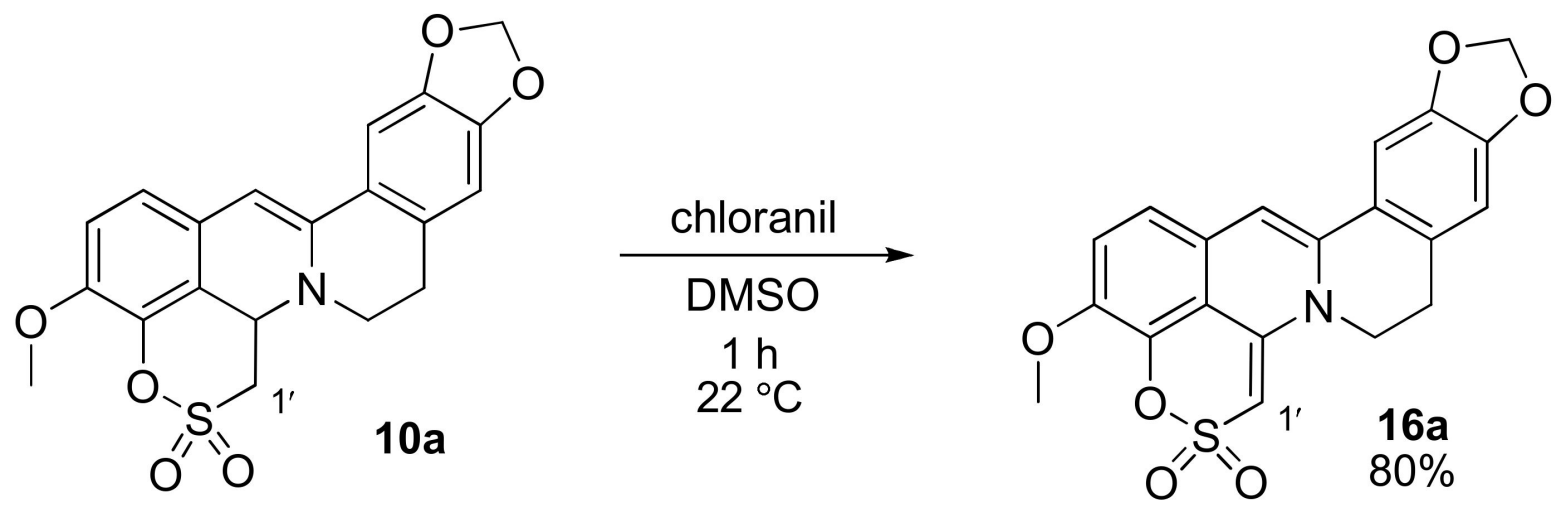
Sultone cycle oxidation with chloranil in DMSO.

Compound **16a** appears to be an oxidation product of sultone **10a** upon DMSO addtion. We carried out a directed oxidation of compound **10a** with chloranil as an oxidizing agent. It was shown that the use of the oxidizer allowed the same reaction to be carried out within 1 h at room temperature (Scheme 9), compound **16a** was isolated from the reaction mixture by precipitation with the addition of water with a yield of 80%.

#### 2.1.4. Synthesis of 12-Bromineberberrubine Derivatives

It is known that the introduction of substituents into the berberine backbone often results in the enhancement of certain biological activities. In particular, it is known that the introduction of bromine at the 12th position of the backbone increased the inhibitory activity of berberine sulfonates against Tdp1 [38]. In order to establish a structure-activity relationship, we synthesized cyclic sultone derivatives from 12-bromberrubine.

**Scheme 10:**
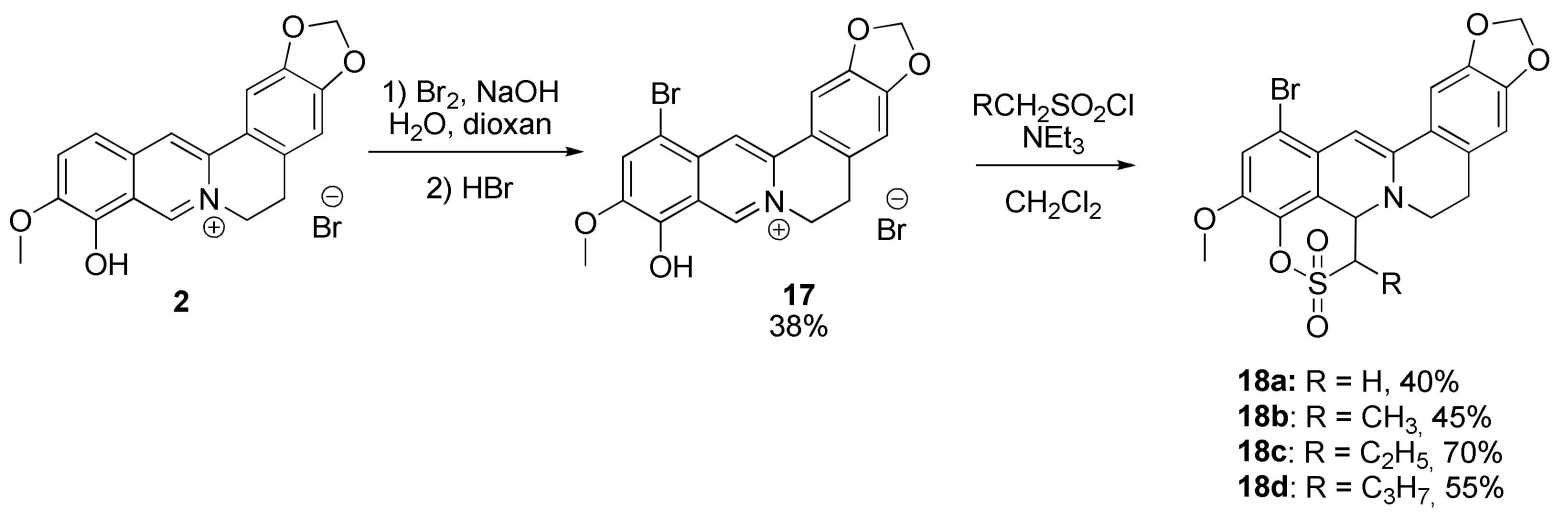
Synthesis of 12-Br-substituted sultones.

We have shown that the reaction of sultones formation from 12-bromoberrubine proceeds similarly to that of berberrubine. 12-Bromoberrubine **17** was obtained from berberrubine **2** by the action of bromine in the presence of alkali in a mixture of dioxane with water according to the procedure described by Nechepurenko et al. [44]. Then, it reacted with sulfochlorides **9a**–**d** by the method we developed for the synthesis of sultones **10a**–**d**, namely, a suspension containing 12-Br-bererrubine **17** with triethylamine was added dropwise to sulfene solutions **15a**–**d**, which led to the formation of sultones **18a–d** in yields from 40% to 70% (Scheme 10). The reaction proceeds at room temperature (22 °C).

**Figure 6:**
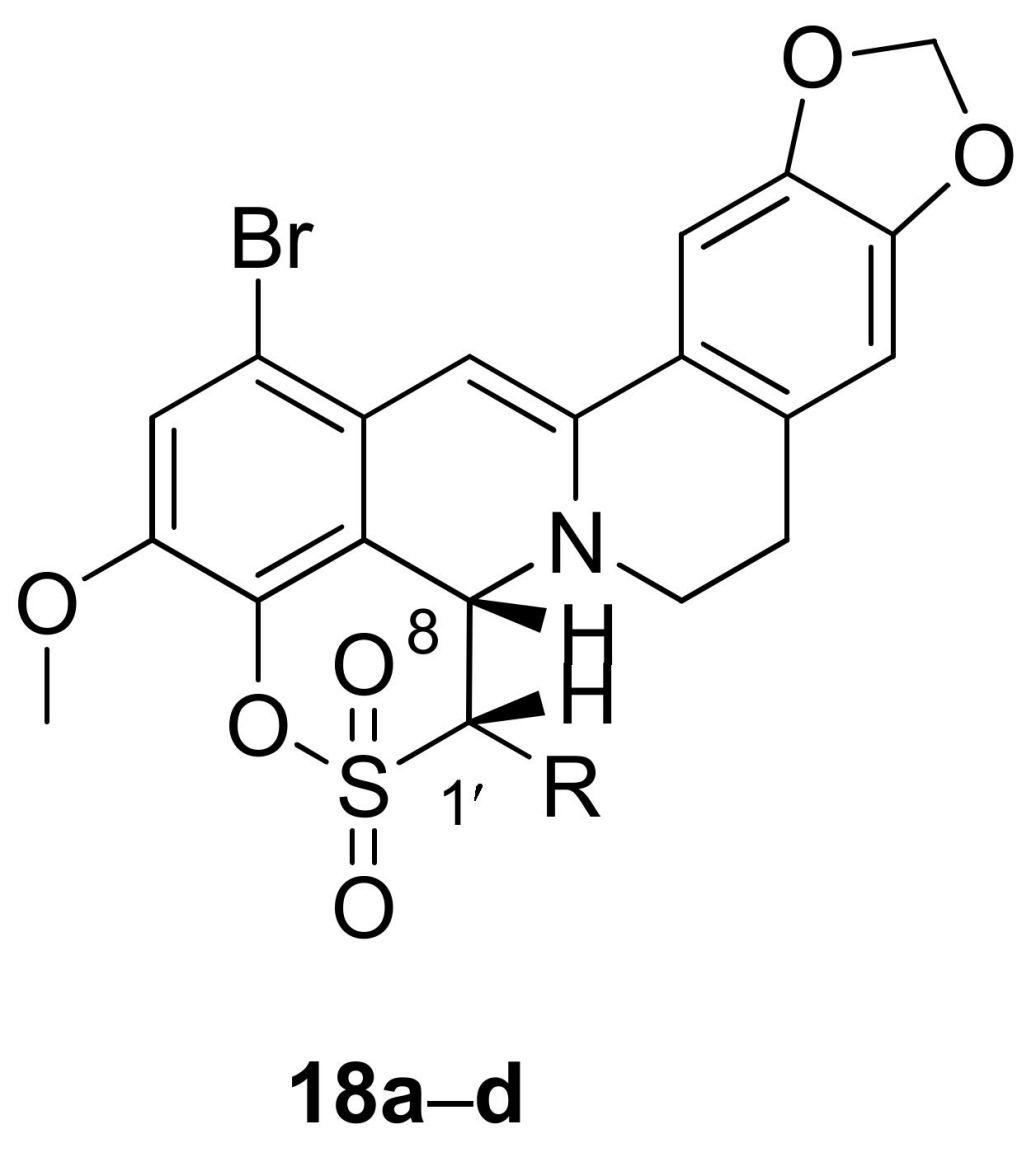
Structure of compounds **18a**–**d**.

In contrast to compounds **10a**–**d**, their brominated analogues **18a**–**d** precipitated during the reaction and were separated from the reaction mixture by filtration in pure form without chromatography. The structure of sultones **18a**–**d** was confirmed by NMR and HR-MS spectroscopies. The dehydrogenation of these compounds occurred under MS conditions, and therefore, [M-2H]^+^ signals were observed in their spectra. In the ^1^H NMR spectra of compounds **18b**–**d**, the proton signal H-8 has a doublet form, the spin–spin interaction constants of protons H-8 and H-1′ vary from 4.60 to 4.79 Hz, indicating a cis-location of protons (Figure 6), as in the case of unbrominated analogues **3b**–**d**.

Thus, we have studied the demethylated reaction at 9-*O*-position berberine and 12-bromberberine derivatives with sulphochlorides and is shown that, in addition to the previously described sulphonates, cyclic sulphonates (sultones) are also formed in the reaction. The study of possible reaction pathways allowed for the selection of reaction conditions that selectively led to sultones in high yields.

### 2.2. Biological Assays

**Figure 7:**
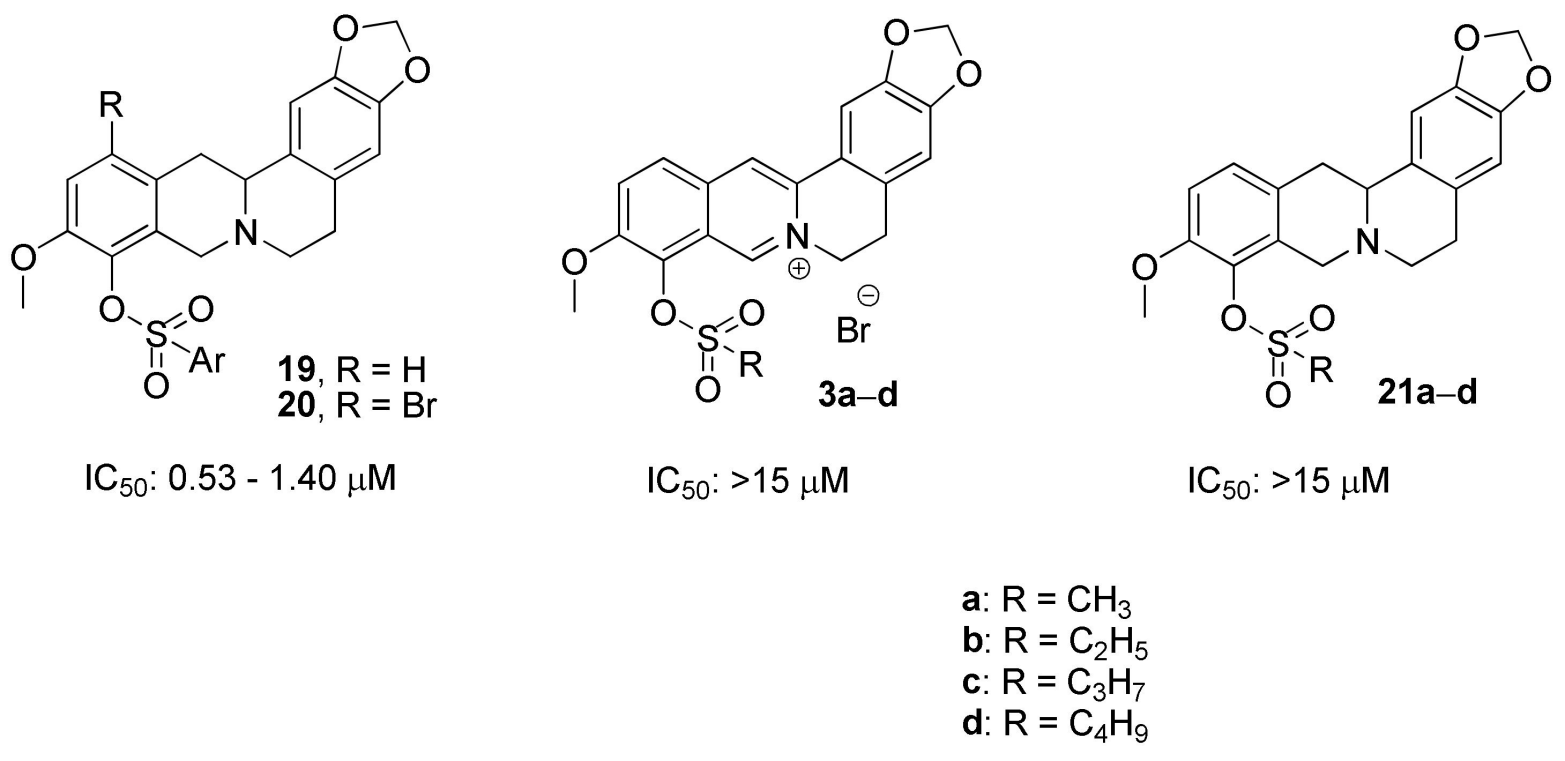
Previously synthesized berberine derivatives tested against Tdp1.

Previously, our group established the inhibitory activity of Tdp1, for 9-*O*-arylsulfonates of berberine (compounds **19**) and enhancement of inhibitory activity after the introduction of bromine at position 12 of the skeleton (compounds **20**). 9-*O*-Alkylsulphonates, both in charged (compounds **3a**–**d**) and reduced form (compounds **21a**–**d**, Figure 7), are not active, with IC_50_ values >15 µM.

In this work, newly synthesized sultones were tested for their inhibition of Tdp1. Tdp1 activity was measured using a fluorescent biosensor as previously described [39]. The biosensor is an oligonucleotide with a fluorophore (FAM) at the 5′-end and a fluorescence quencher (BHQ1) at the 3′-end. Due to the activity of the enzyme, the quencher is removed, which leads to an increase in the fluorescence intensity. Inhibitors prevent the removal of the quencher, thus reducing the fluorescence intensity. The results are shown in Table 1.

All new compounds with the cyclic sulfone fragment show inhibitory activity in the micromole or submicromolar range. Introduction of bromine atom in a molecule led to an increase in activity. As for the substituent in the sultone ring, there is no strict correlation between the carbon chain length and inhibitory activity. Inhibitory activity, comparable to that of sultone **10a,** was found for its dehydrogenated analog compound **16a** (IC_50_ 2.50 ± 0.40 µM).

**Figure 8:**
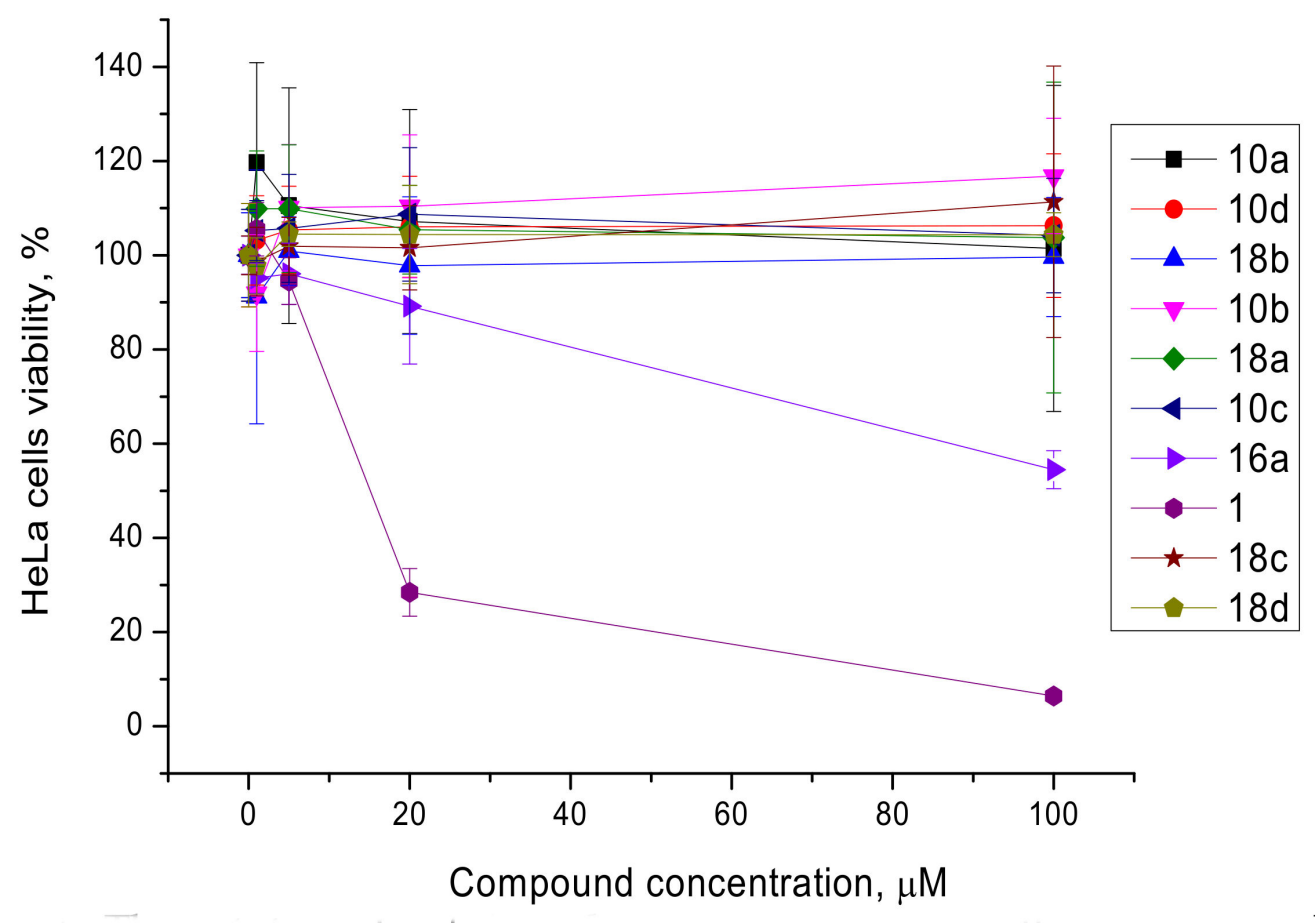
The cytotoxicity of the sultones against HeLa cells, as measured by theEZ4U test.

All compounds have no intrinsic toxicity in the concentration range up to 100 μM (Figure 8) in HeLa cells, with the exception of **16a** and berberine, which are toxic at concentrations above 20 μM. **16a** is slightly toxic (CC_50_ ~ 100 μM) and differs from non-toxic **10a** in the presence of a double bond in the sultone cycle. The original berberine **1** is toxic to HeLa cells with a cytotoxic concentration (CC_50_) of 20 μM, which is consistent with the literature data [45].

**Figure 9:**
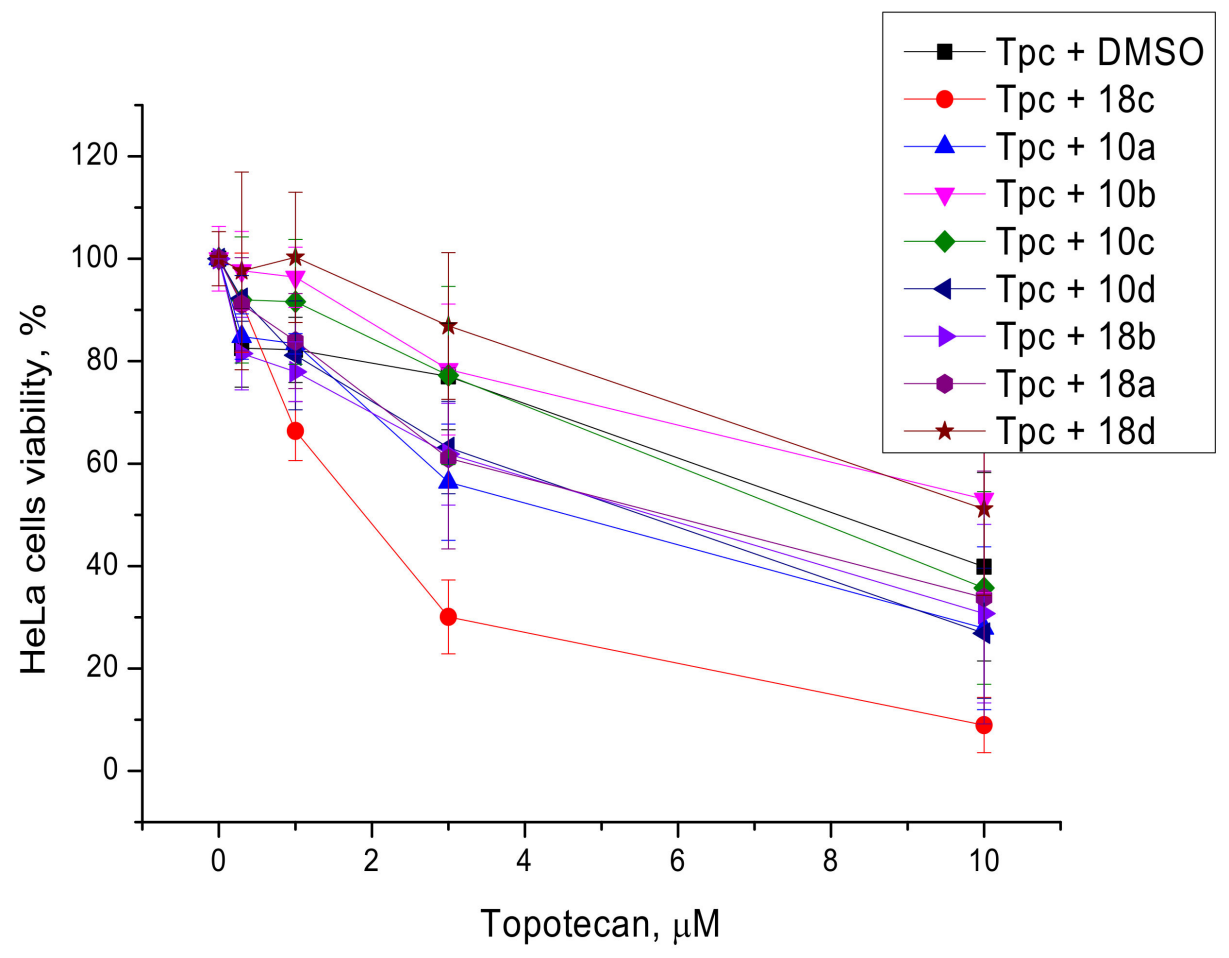
The influence of the berberrubine sultones at 20 μM on topotecan cytotoxicity. Error bars show standard deviations.

As already mentioned, we have previously shown that various Tdp1 inhibitors enhanced the cytotoxic and antitumor effects of topotecan [21,22,23,24,25,26,27,28,29,30]. In this work, we also tested the ability of the berberrubine sultones to sensitize cells to the action of topotecan. For this, we chose a non-toxic concentration of Tdp1 inhibitors at 20 μM and varied the concentrations of topotecan. As shown in Figure 9, only compound **18c** exhibited the most pronounced and statistically significant (Mann—Whitney U-test, *p* = 0.05) sensitizing effect.

Effective inhibition of Tdp1 and low toxicity make the berberrubine sultones promising candidates for the further development of tumor cell sensitizers to anticancer drugs.

### 2.3. Molecular Modelling

**Figure 10:**
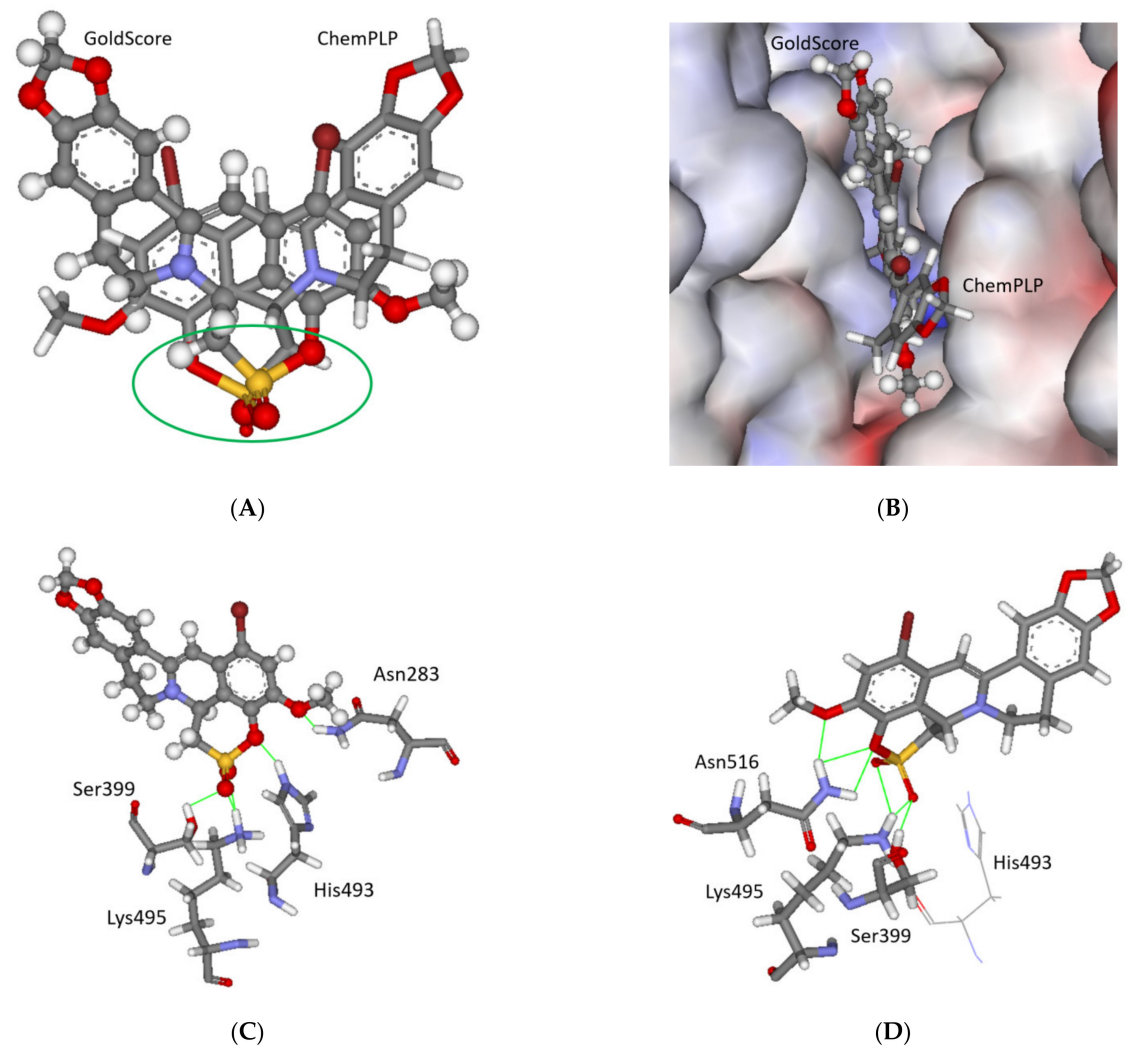
The docked configuration of **18a** in the binding site of Tdp1 as predicted by the GoldScore (ball and stick) and ChemPLP (stick) scoring functions. (**A**) The docked overlaid conformations for the GoldScore and ChemPLP predictions. The hydrogen bonding sulfonate moiety is circled in green. (**B**) The protein surface is rendered; blue depicts regions with a partial positive charge on the surface; red depicts regions with a partial negative charge and grey shows neutral areas. The ligand occupies the binding pocket, blocking access to it in both predicted configurations. (**C**,**D**) Hydrogen bonds are shown as green lines between the ligand (**C**), as predicted by GoldScore with amino acids Asn283, Ser399, His493 and Lys495; (**D**) was predicted by ChemPLP with amino acids Ser399, Lys495 and Asn516.

Berberine derivatives were docked into the binding site of the Tdp1 structure (PDB ID: 6DIE, resolution 1.78 Å) [41], with three water molecules retained (HOH 814, 821 and 1078). It has been shown that keeping these crystalline water molecules improves the prediction quality of the docking scaffold (see the Methodology section for further information) [25]. The binding predictions of the four scoring functions used are given in Table S1; all the ligands show reasonable scores. When the predicted configurations were analyzed, two main binding modes emerged, depending on the scoring function used and the type of derivative. Both configurations involve hydrogen bonding via the 1,4-butanesultone ring. In order to check which conformation is more stable, 10 ps molecular dynamics (MD) runs at 1000 K were conducted with derivative **18a**. It is predicted to be bound to the protein in both conformations depending on the scoring function. The results indicate that both conformations are stable for the 10 ps run, e.g., the hydrogen bonding networks between the ligand and Tdp1 were not broken. Thus, it can be concluded that both conformations are viable, and they are shown in Figure 10.

In both predicted poses for **18a,** the sulfonate ring moiety plays a major role in the hydrogen bonding to the Tdp1 enzyme. Both form hydrogen bonds to Ser399 and Lys495 via the sulfonyl moiety. His493 is predicted to bind to the ring forming oxygen for GoldScore, but to Asn516 for ChemPLP. In both scenarios, the methoxy group is involved in hydrogen bonding to the enzyme, but according to the MD runs, it is not very stable and often breaks. In general, both predicted conformations are plausible, with a good fit into the binding site, blocking access to His493, one of the crucial amino acid residues for catalytic activity. Interestingly, the binding mode of the berberine derivatives presented in this study differs from previously reported berberine derivatives for Tdp1 inhibition [38]. This can be explained by the lack of the 1,4-butanesultone ring in these berberine ligands, which is clearly the main hydrogen bonding motive in the derivatives presented here. The driving force of the previously reported ligands is most likely a lone pair—π stacking with a fluorinated phenyl moiety absent in the ligands in this study. Additionally, derivatives **21a** and **21b**, which do not have the 1,4-butanesultone ring scaffold and are inactive at >15 µM, are not predicted to have a specific binding mode, each scoring function offers a different solution, further strengthening the argument for the importance of this ring motif (see Figure 7). Finally, the bromine moiety in the previously reported ligands is predicted to form weak hydrogen bonds with His493 [38]; this is not seen for the berberine series presented here, where the bromine is pointing towards the aqueous phase and its effect on increased binding can be explained in terms of solvent entropic effects (see the Chemical Space Section).

The co-crystalized ligand benzene-1,2,4-tricarboxylic acid forms hydrogen bonds with the side chains of Ser399, His493 and Lys495 [41], i.e., the same amino acid residues as **18a**. In general, His263 [35,46], His493 [38,47] or both [33,48] are usually predicted to have direct hydrogen bonding to the active ligands, as well as Lys495 [25,30], irrespective of the ligand molecular type used. This is easily understood since these three amino acid residues act as the catalytic scaffold for disengaging DNA from Top1. 

### 2.4. Chemical Space

**Figure 11:**
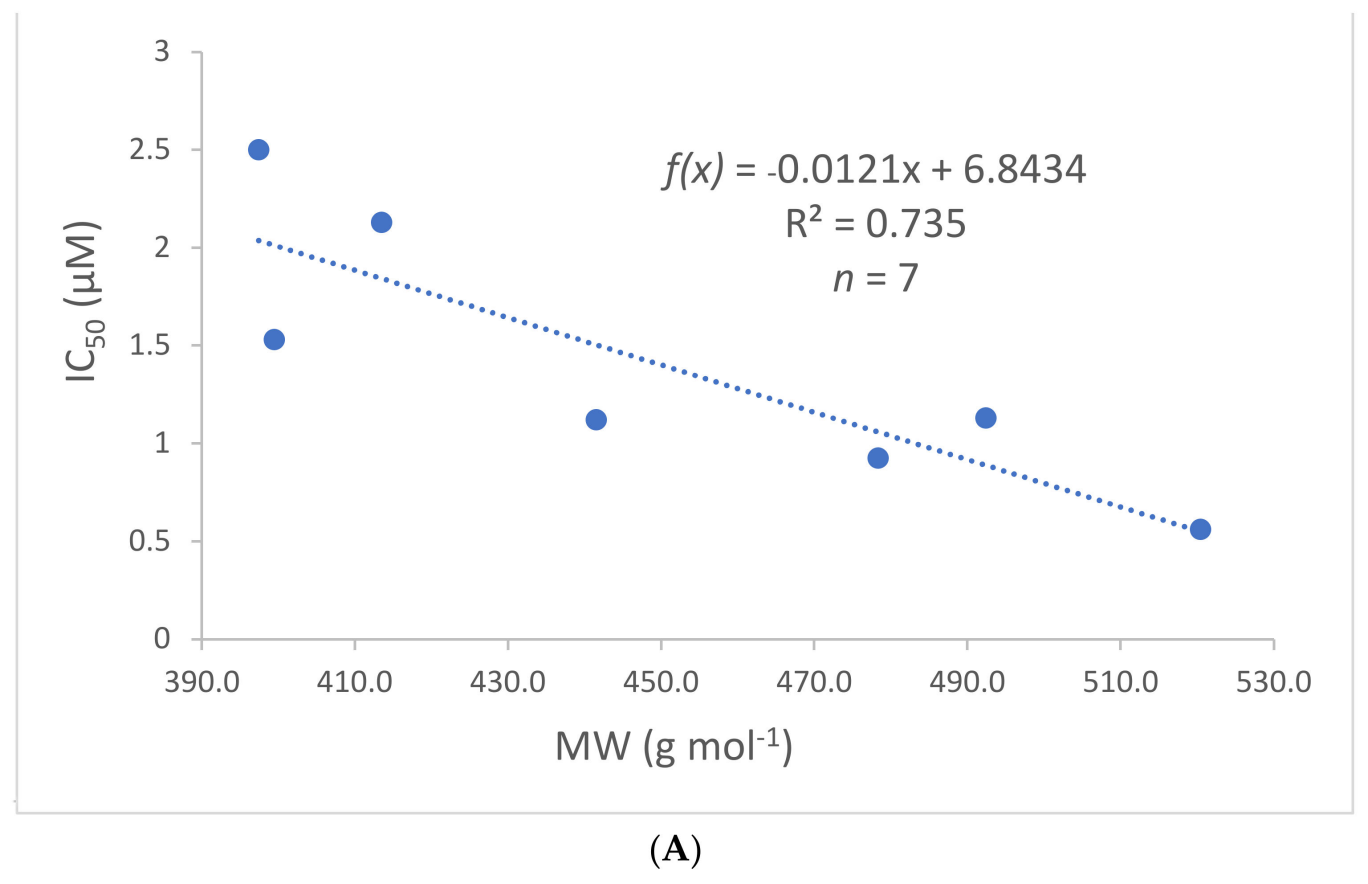

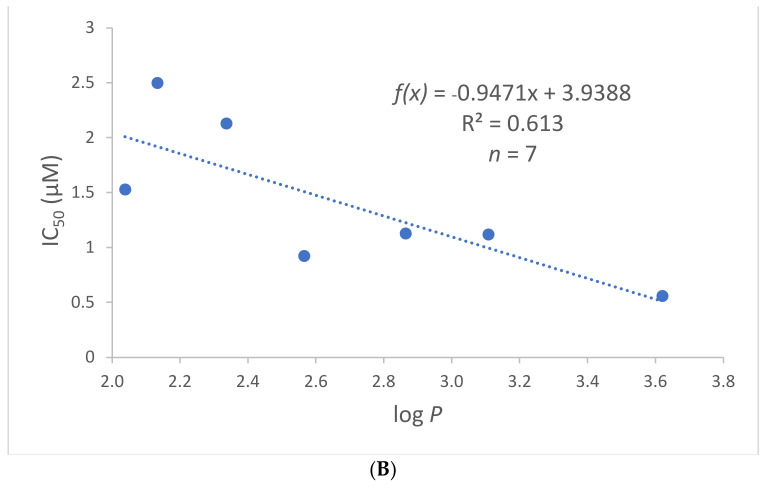
The correlation of the IC_50_ values of the ligands with MW (**A**) and log *P* (**B**). A very good correlation is seen for both molecular descriptors. Derivative **10c** is an outlier and not included. Additionally, derivatives **21a** and **21b** are omitted since they are inactive.

The calculated molecular descriptors MW (molecular weight), log *P* (water-octanol partition coefficient), HD (hydrogen bond donors), HA (hydrogen bond acceptors), PSA (polar surface area) and RB (rotatable bonds)) are given in Table S2. The MW of the ligands lie in the range of 399.4 and 520.4 g mol^−1^, falling into drug-like chemical space and for two derivatives in Known Drug Space (KDS). Obviously, the brominated derivatives have higher MW. The Log P values are very modest, lying between 2.0 and 3.6; in the lead-like and drug-like chemical spaces like the other four descriptors (for the definition of lead-like, drug-like and Known Drug Space regions see Reference [49] and Table S3). When the activity of the ligands is plotted against the molecular descriptors strong correlation is seen with both MW and log *P,* as shown in Figure 11. Derivative **10c** is an outlier and is omitted, as well as derivatives **21a** and **21b**, which are practically inactive.

As can be seen in Figure 11, a strong correlation is seen with both MW and log *P* with higher numbers favoring better IC_50_ values. The HD and HA do not correlate with the activity, but PSA (R^2^—0.257) and RB (R^2^—0.324) do to some extent. It is therefore clear from these results that increased MW and log *P* favor improved binding to Tdp1. The same trend is seen for deoxycholic acid steroid derivatives, which are also excellent Tdp1 inhibitors [48].

The Known Drug Indexes (KDIs) for the ligands were calculated to gauge the balance of the molecular descriptors (MW, log *P*, HD, HA, PSA and RB). This method is based on the analysis of drugs in clinical use, i.e., the statistical distribution of each descriptor is fitted to a Gaussian function and normalized to 1, resulting in a weighted index. Both the summation of the indexes (*KDI_2a_*) and multiplication (*KDI_2b_*) methods were used [50], as shown for *KDI_2a_* in Equation (1) and for *KDI_2b_* in Equation (2); the numerical results are given in Table S2 in the Supplementary Materials.

*KDI_2a_ = I_MW_ + I_log P_ + I_HD_ + I_HA_ + I_RB_ + I_PSA_* (1)

*KDI_2b_ = I_MW_ × I_log P_ × I_HD_ × I_HA_ × I_RB_ × I_PSA_* (2)

**Figure 12:**
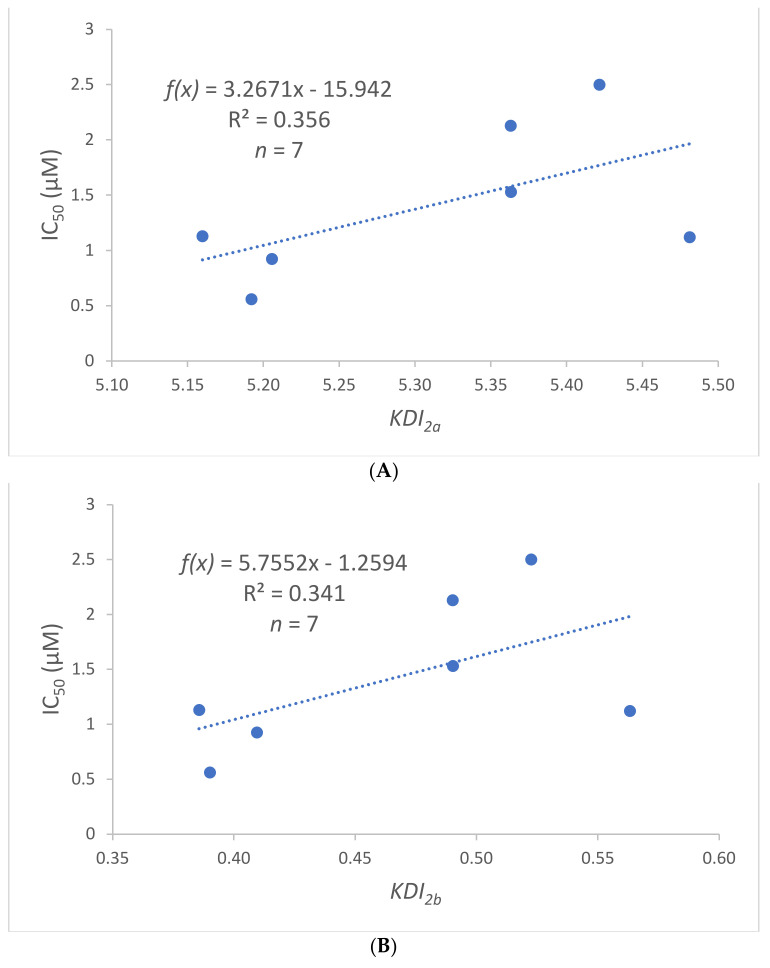
The correlations between *KDI_2a_* (**A**) and *KDI_2b_* (**B**) with their corresponding IC_50_ values. Smaller KDI values favor increased activity.

The *KDI_2a_* values for the ligands range from 5.16 to 5.57 with a theoretical maximum of 6 and the average of 4.08 (±1.27) for known drugs. These values are very good, since most of the descriptors lie within the lead and drug-like boundaries of chemical space. *KDI_2b_* range from 0.39 to 0.62, with a theoretical maximum of 1 and with a KDS average of 0.18 (±0.20). Again, very good values are obtained for the ligands even though the *KDI_2b_* index is more sensitive than *KDI_2a_* to outliers, since multiplication of small numbers leads to smaller numbers. KDI indexes were plotted against the IC_50_ values with the same ligands as for MW and log *P* and the results are shown in Figure 12.

For both KDI indexes, lower values result in better activity; more lipophilic and larger compounds are pushed into the catalytic cite by solvent entropic effects. The same trend is seen for deoxycholic acid steroid derivatives [48]. Therefore, the addition of bromine to the ligands does not contribute to more bonding between the ligands and Tdp1 (see modelling section), rather it increases the entropic push of the ligand into the binding pocket. 

## 3. Materials and Methods

### 3.1. Chemistry

General information. Reagents (berberine chloride, alkyl sulfochlorides, triethylamine, sodium borohydride, chloranil) were purchased from commercial suppliers (Sigma-Aldrich, Acros, TCI) and used as received. Berberrubine hydrobromide and 12-bromoberberrubine were synthesized according to previously reported methods [44,51]. Solvents (dichloromethane, DMSO) were used after distillation. ^1^H and ^13^C NMR were recorded at Bruker DRX-500 apparatus at 125.76 MHz (^13^C), Bruker AV-300 apparatus at 300.13 MHz (^1^H) and 75.47 MHz (^13^C), Bruker AV-400 apparatus at 400.13 MHz (^1^H) and 100.61 (^13^C), Bruker Avance—III 600 apparatusat 600.30 MHz (^1^H) and 150.95 MHz (^13^C), Jin Hz; structure determinations by analyzing the ^1^H NMR spectra, including ^1^H-^1^H double resonance spectra and ^1^H-^1^H 2D homonuclear correlation, J-modulated ^13^C NMR spectra (JMOD), and ^13^C-^1^H 2D heteronuclear correlation with one-bond (C-H COSY, ^1^J(C,H) = 160 Hz, HSQC, ^1^J(C,H) = 145 Hz) and long-range spin-spin coupling constants (COLOC, ^2,3^J(C,H) = 10 Hz, HMBC, ^2,3^J(C,H) = 7 Hz). HR-MS: DFS Thermo Scientific spectrometer in a full scan mode (15–500 *m*/*z*, 70 eV electron impact ionization, direct sample administration).

Spectral and analytical investigations were carried out at the Multi-Access Chemical Research Center of Siberian Branch of Russian Academy of Sciences. All product yields are given for pure compounds.

#### 3.1.1. General Procedure for the Synthesis of Sultones **10a**–**d**

The stirred solution of sulfochloride (1.5 mmol) with triethylamine (1.5 mmol) in 25 mL of dichloromethane suspension of berberrubine hydrobromide (1 mmol) with triethylamine (2.5 mmol) in 3 mL of dichloromethane was added dropwise at room temperature (22–24 °C). After completion, the reaction (monitored by TLC) mixture was washed with water, organic layer dried with MgSO_4_. After purification by column chromatography (eluent—dichloromethane: methanol, sorbent-silica gel), the products **10a**–**d** were obtained.

*19-Methoxy-8,10,21-trioxa-22λ⁶-thia-2-azahexacyclo[14.7.1.0^2^,¹⁴.0⁵,¹^3^.0⁷,¹¹.0^2^⁰,^2^⁴]tetracosa-5,7(11),12,14,16,18,20(24)-heptaene-22,22-dione* (**10a**). Yield: 70%. HR-MS, m/z: found 399.0768. Calculated for (C_20_H_17_O_6_N_1_^32^S_1_)^+^: 399.0771. NMR ^1^H (400 MHz, DMSO-d_6_, δ, ppm): 2.73 (3H, m, H-3, H-4), 3.28–3.32 (1H, m, H-3), 3.80 (3H, s, OCH_3_), 4.11 (1H, t H-1), 4.56 (1H, d.d, J_1_ = 5.64 Hz, J_2_ = 11.89 Hz, H-23), 4.69 (1H, d.d, J_1_ = 5.64 Hz, J_2_ = 13.03 Hz, H-23), 6.01 (2H, d.d, J_1_ = 9.60 Hz, J_2_ = 0.88 Hz, H-9), 6.37 (1H, s, H-6), 6.78 (1H, s, H-12), 6.90 (1H, d, J = 8.40 Hz, H-17), 7.07 (1H, d, J = 8.40 Hz, H-18), 7.35 (1H, s, H-15). NMR ^13^C (100 MHz, CDCl_3_, δ, ppm): 29.37 (C-4), 44.47 (C-3), 48.46 (C-23), 55.78, 56.29 (C-1), (OCH_3_), 98.32 (C-15), 101.00 (C-9), 103.67 (C-12), 107.36 (C-16), 113.08 (C-18), 113.38 (C-24), 119.91 (C-17), 123.66 (C-13), 126.86 (C-16), 128.27 (C-5), 137.67 (C-14), 141.72 (C-20), 146.10 (C-7), 146.80 (C-11), 147.62 (C-19).

*19-Methoxy-23-methyl-8,10,21-trioxa-22λ⁶-thia-2-azahexacyclo[14.7.1.0^2^,¹⁴.0⁵,¹^3^.0⁷,¹¹.0^2^⁰,^2^⁴]tetracosa-5,7(11),12,14,16,18,20(24)-heptaene-22,22-dione* (**10b**). Yield: 65%. HR-MS, m/z: found 413.0924. Calculated for (C_21_H_19_O_6_N_1_^32^S_1_)^+^: 413.0928. NMR ^1^H (600 MHz, DMSO-d_6_, δ, ppm, J/Hz): 1.37 (3H, d, J = 6.69 Hz, H-1′), 2.69–2.84 (3H, m, H-3, H-4), 3.23–3.29 (1H, m, H-3), 3.80 (3H, s, OCH_3_), 4.71 (1H, m, H-23), 4.90 (1H, d, J = 4.56 Hz, H-1), 4.99 (1H, s, J = 4.74, H-1′), 6.01 (2H, d.d, J_1_ = 9.12 Hz, J_2_ = 1.00 Hz, H-9), 6.29 (1H, s, H-12), 6.76 (1H, s, H-6), 6.87 (1H, d, J = 8.44 Hz, H-17), 7.05 (1H, d, J = 8.40 Hz, H-18), 7.37 (1H, s, H-15). NMR ^13^C (100 MHz, DMSO-d_6_, δ, ppm): 9.73 (C-1′), 28.84 (C-4), 43.93 (C-3), 50.93, 56.03, 59.24 (OCH_3_, C-1, C-23), 96.88 (C-15), 101.13 (C-9), 103.83 (C-12), 107.69 (C-6), 110.92 (C-24), 113.37 (C-18), 119.96 (C-17), 123.52 (C-13), 127.48 (C-16), 129.02 (C-5), 136.67 (C-14), 141.19 (C-20), 145.39 (C-7), 146.60 (C-11), 147.34 (C-19).

*23-Ethyl-19-methoxy-8,10,21-trioxa-22λ⁶-thia-2-azahexacyclo[14.7.1.0^2^,¹⁴.0⁵,¹^3^.0⁷,¹¹.0^2^⁰,^2^⁴]tetracosa-5,7(11),12,14,16,18,20(24)-heptaene-22,22-dione* (**10c**). Yield: 68%. HR-MS, m/z: found 427.1068. Calculated for (C_22_H_21_O_6_N_1_^32^S_1_)^+^: 427.1084. NMR ^1^H (400 MHz, DMSO-d_6_, δ, ppm): 1.17 (3H, t, J = 7.58 Hz, H-2′), 1.57–1.66 (1H, m, H-1′), 2.02–2.09 (1H, m, H-1′), 2.76–2.83 (3H, m, H-3, H-4), 3.16–3.20 (1H, m, H-3), 3.79 (3H, s, OCH_3_), 4.48–4.53 (1H, m, H-23), 4.97 (1H, d, J = 4.69 Hz, H-1), 6.02 (2H, d, J = 6.09 Hz, H-9), 6.23 (1H, s, H-6), 6.77 (1H, s, H-12), 6.86 (1H, d, J = 8.57 Hz, H-17), 7.04 (1H, d, J = 8.57 Hz, H-18), 7.36 (1H, s, H-15). NMR ^13^C (100 MHz, DMSO-d_6_, δ, ppm): 12.04 (C-2′), 18.15 (C-1′), 28.72 (C-4), 44.19 (C-3), 56.04 (OCH_3_), 57.72, 59.70 (C-1), (C-23), 96.04 (C-15), 101.11 (C-9), 103.86 (C-12), 107.69 (C-6), 112.00, 123.55, 127.17, 128,90 (C-5, C-24, C-16, C-13), 113.43 (C-18), 119.98 (C-17), 136.58 (C-14), 140.77 (C-20), 145.42 (C-7), 146.58 (C-11), 147.34 (C-19).

*19-Methoxy-23-propyl-8,10,21-trioxa-22λ⁶-thia-2-azahexacyclo[14.7.1.0^2^,¹⁴.0⁵,¹^3^.0⁷,¹¹.0^2^⁰,^2^⁴]tetracosa-5,7(11),12,14,16,18,20(24)-heptaene-22,22-dione* (**10d**). Yield: 64%. HR-MS, m/z: found 441.1220. Calculated for (C_23_H_23_O_6_N_1_^32^S_1_)^+^: 441.1241. NMR ^1^H (400 MHz, DMSO-d_6_, δ, ppm): 0.90 (3H, t, J = 6.89 Hz, H-3′), 1.55–1.74 (3H, m, H-2′, H-1′), 1.91–1.99 (1H, m, H-1′), 2.76–2.83 (3H, m, H-3, H-4), 3.17–3.22 (1H, m, H-3), 3.79 (3H, s, OCH3), 4.57 (1H, m, H-23), 5.01 (1H, d, J = 4.75 Hz, H-1), 6.01 (2H, d.d, J_1_ = 4.67 Hz, J_2_ = 0.77 Hz, C-9), 6.22 (1H, s, H-6), 6.77 (1H, s, H-12), 6.86 (1H, d, J = 8.42 Hz, H-17), 7.04 (1H, d, J = 8.42 Hz, H-18), 7.36 (1H, s, H-15). NMR ^13^C (100 MHz, DMSO-d_6_, δ, ppm): 13.89 (C-3′), 20.24 (C-2′), 26.76 (C-1′), 28.70 (C-4), 44.19 (C-3), 56.00, 56.04, 59.70 (OCH_3_, C-1, C-23), 95.93 (C-15), 101.11 (C-9), 103.83 (C-12), 107.69 (C-6), 111.84, 123.52, 127.09, 128.87 (C-5, C-24, C-16, C-13), 113.37 (C-18), 119.97 (C-17), 136.57 (C-14), 140.68 (C-20), 145.39 (C-7), 146.57 (C-11), 147.33 (C-19).

*19-Methoxy-8,10,21-trioxa-22λ⁶-thia-2-azahexacyclo[14.7.1.0^2^,¹⁴.0⁵,¹^3^.0⁷,¹¹.0^2^⁰,^2^⁴]tetracosa-1(23),5,7(11),12,14,16,18,20(24)-octaene-22,22-dione* (**16a**). The suspension of compound **10a** (399 mg, 1 mmol) in dimethyl sulfoxide (10 mL) and 246 mg (1 mmol) of chloranil was added in one portion. The reaction mixture was stirred at room temperature for 1 h. After that cold water was added and we observed the greenish-yellow precipitate formation. The precipitate was filtered, washed with cold water and air-dried. 316 mg of product **16a** was obtained. Yield: 80%. HRMS, m/z: found 397.0618. Calculated for (C_20_H_15_O_6_N_1_^32^S_1_)^+^: 397.0615. NMR ^1^H (400 MHz, DMSO-d_6_, δ, ppm): 2.94 (2H, t, J = 6.23 Hz, H-4), 3.68 (2H, t, J = 6.23 Hz, H-3), 3.91 (3H, s, OCH_3_), 6.07 (1H, s, H-23), 6.08 (2H, s, H-9), 6.95 (1H, s, H-15 *), 7.03 (1H, s, H-12 *), 7.36 (1H, d, J = 8.70 Hz, H-18), 7.42 (1H, s, H-6 *), 7.52 (1H, d, J = 8.70 Hz, H-17). NMR 13C (100 MHz, DMSO-d_6_, δ, ppm): 26.96 (C-4), 43.23 (C-3), 56.58 (OCH_3_), 83.79 (C-23), 101.40 (C-9), 101.45 (C-15), 104.89 (C-12), 107.60 (C-6), 111.88 (C-24), 118.26 (C-18), 121.76 (C-17), 122.48, 126.14, 128.98, 135.25 (C-5, C-16, C-14, C-13), 144.00 (C-1), 145.66, 145.83, 146.96 (C-7, C-11, C-20), 148.29 (C-19). Signals marked with * can be swapped.

#### 3.1.2. General Procedure for the Synthesis of Sultones **18a**–**d**

The suspension of 0.22 mmol 12-bromoberberrubine in 3 mL of dichloromethane 0.091 mmol trimethylamine was added dropwise under stirring at room temperature (24 °C). The resulting solution (solution 1) was left to stir for 5 min. The solution of 0.34 mmol of sulfochloride in 25 mL of methylene chloride was added 0.34 mmol of triethylamine (solution 2). Solution 1 was added to solution 2 dropwise. After 3 h, the resulting yellow precipitate was filtered off, washed with 10 mL cold methylene chloride and the pure product **18a**–**d** was isolated.

*17-Bromo-19-methoxy-8,10,21-trioxa-22λ⁶-thia-2-azahexacyclo[14.7.1.0^2^,¹⁴.0⁵,¹^3^.0⁷,¹¹.0^2^⁰,^2^⁴]tetracosa-5,7(11),12,14,16,18,20(24)-heptaene-22,22-dione* (**18a**). Yield: 40%. HR-MS, m/z: found for (C_20_H_16_O_6_N_1_^79^Br_1_^32^S_1_-2H)^+^: 474.9723. Calculated for (C_20_H_16_O_6_N_1_^79^Br_1_^32^S_1_)^+^: 476.9876. NMR ^1^H (400 MHz, DMSO-d_6_, δ, ppm): 2.64–2.73 (1H, m, H-4), 2.78–2.86 (2H, m, H-3, H-4), 3.28 (1H, m, H-3), 3.84 (3H, s, OCH_3_), 4.24 (1H, t, J = 12.37 Hz, H-1), 4.56 (1H, d.d, J_1_ = 5.50 Hz, J_2_ = 11.50 Hz, H-23), 4.69 (1H, d.d, J_1_ = 5.50 Hz, J_2_ = 12.69 Hz, H-23), 6.02 (1H, s, H-9), 6.06 (1H, s, H-9), 6.25 (1H, s, H-6), 6.82 (1H, s, H-12), 7.32 (1H, s, J = 8.40 Hz, H-18), 7.36 (1H, s, H-15). NMR ^13^C (100 MHz, DMSO-d_6_, δ, d.d.): 29.24 (C-4), 44.13 (C-3), 47.44 (C-23), 56.19, 57.23 (C-1), (OCH_3_), 96.42 (C-15), 101.85 (C-9), 104.44 (C-12), 108.29 (C-6), 113.59 (C-17), 115.03 (C-18), 117.53 (C-24), 123.83 (C-13), 126.59 (C-5), 130.05 (C-16), 137.37 (C-14), 144.88 (C-20), 146.30 (C-7), 147.34 (C-11), 148.45 (C-19).

*17-Bromo-19-methoxy-23-methyl-8,10,21-trioxa-22λ⁶-thia-2-azahexacyclo[14.7.1.0^2^,¹⁴.0⁵,¹^3^.0⁷,¹¹.0^2^⁰,^2^⁴]tetracosa-5,7(11),12,14,16,18,20(24)-heptaene-22,22-dione* (**18b**). Yield: 45%. HR-MS, m/z: found for (C_21_H_18_O_6_N_1_^79^Br_1_^32^S_1_-2H)^+^:∙488.9878. Calculated for (C_20_H_18_O_6_N_1_^79^Br_1_^32^S_1_)^+^: 491.0033. NMR ^1^H (400 MHz, DMSO-d_6_, δ, ppm): 1.38 (3H, d, J = 6.75 Hz, H-1′), 2.77–2.84 (3H, m, H-4, H-3), 3.22–3.27 (1H, m, H-3), 3.83 (3H, s, OCH_3_), 4.73–4.80 (1H, m, H-23), 5.06 (1H, d, J = 4.71 Hz, H-1), 6.03 (1H, s, H-9), 6.06 (1H, s, H-9), 6.13 (1H, s, H-6), 6.82 (1H, s, H-12), 7.29 (1H, s, H-18), 7.34 (1H, s, H-15). NMR ^13^C (100 MHz, DMSO-d_6_, δ, ppm): 9.65 (C-1′), 29.13 (C-4), 43.86 (C-3), 52.56 (C-23), 56.14, 59.71 (C-1), (OCH_3_), 95.09 (C-15), 100.91 (C-9), 103.98 (C-12), 107.19 (C-6), 113.05, 114.17 (C-17), (C-24), 116.68 (C-18), 123.34 (C-13), 126.14 (C-5), 128.42 (C-16), 136.29 (C-14), 142.34 (C-20), 145.85 (C-7), 146.70 (C-11), 147.80 (C-19).

*17-Bromo-23-ethyl-19-methoxy-8,10,21-trioxa-22λ⁶-thia-2-azahexacyclo[14.7.1.0^2^,¹⁴.0⁵,¹^3^.0⁷,¹¹.0^2^⁰,^2^⁴]tetracosa-5,7(11),12,14,16,18,20(24)-heptaene-22,22-dione* (**18c**). Yield: 70%. HR-MS, m/z: found for (C_22_H_20_O_6_N_1_^79^Br_1_^32^S_1_-2H)^+^:∙503.0033. Calculated for (C_22_H_20_O_6_N_1_^79^Br_1_^32^S_1_)^+^: 505.0189. NMR ^1^H (400 MHz, DMSO-d_6_, δ, ppm): 1.15 (3H, t, J = 7.39 Hz, H-2′), 1.54–1.66 (1H, m, H-1′), 1.96–2.06 (1H, m, H-1′), 2.78–2.93 (3H, m, H-4, H-3), 3.14–3.20 (1H, m, H-3), 3.82 (3H, s, OCH_3_), 4.50–4.56 (1H, m, H-23), 5.17 (1H, d, J = 4.60 Hz, H-1), 6.02 (1H, s, H-9), 6.03 (1H, s, H-9), 6.05 (1H, s, H-6), 6.82 (1H, s, H-12), 7.30 (1H, s, H-18), 7.33 (1H, s, H-15). NMR ^13^C (100 MHz, DMSO-d_6_, δ, ppm): 12.33 (C-2′), 18.69 (C-1′), 28.89 (C-4), 44.16 (C-3), 56.80, 59.02, 59.95 (C-1), (C-23), (OCH_3_), 93.23 (C-15), 101.62 (C-9), 104.15 (C-12), 108.13 (C-6), 113.82, 114.47 (C-17), (C-24), 117.35 (C-18), 123.39 (C-13), 126.11 (C-5), 129.98 (C-16), 136.19 (C-14), 143.19 (C-20), 145.98 (C-7), 146.99 (C-11), 148.22 (C-19).

*17-Bromo-19-methoxy-23-propyl-8,10,21-trioxa-22λ⁶-thia-2-azahexacyclo[14.7.1.0^2^,¹⁴.0⁵,¹^3^.0⁷,¹¹.0^2^⁰,^2^⁴]tetracosa-5,7(11),12,14,16,18,20(24)-heptaene-22,22-dione* (**18d**). Yield: 55%. HR-MS, m/z: found for (C_23_H_22_O_6_N_1_^79^Br_1_^32^S_1_-2H)^+^: 517.0189. Calculated for (C_23_H_22_O_6_N_1_^79^Br_1_^32^S_1_)^+^: 519.0346. NMR ^1^H (400 MHz, DMSO-d_6_, δ, ppm): 0.89 (3H, t, J = 7.12 Hz, H-3′), 1.51–1.69 (2H, m, H-2′), 1.64–1.73 (1H, m, H-1′), 1.87–1.95 (1H, m, H-1′), 2.78–2.92 (3H, m, H-4, H-3), 3.14–3.21 (1H, m, H-3), 3.82 (3H, s, OCH_3_), 4.57–4.62 (1H, m, H-23), 5.19 (1H, d, J = 4.69 Hz, H-1), 6.01 (1H, s, H-9), 6.03 (1H, s, H-9), 6.05 (1H, s, H-6), 6.82 (1H, s, H-12), 7.26 (1H, s, H-18), 7.31 (1H, s, H-15). NMR ^13^C (100 MHz, DMSO-d_6_, δ, ppm): 13.87 (C-3′), 20.30 (C-2′), 26.99 (C-1′), 28.60 (C-4), 43.93 (C-3), 56.66, 57.08, 59.67 (C-1), (C-23), (OCH_3_), 92.97 (C-15), 101.40 (C-9), 103.86 (C-12), 107.87 (C-6), 113.68, 114.13 (C-17), (C-24), 117.18 (C-18), 123.19 (C-13), 125.87 (C-5), 129.70 (C-16), 136.01 (C-14), 142.86 (C-20), 145.78 (C-7), 146.72 (C-11), 147.95 (C-19).

### 3.2. Biology

#### 3.2.1. Detection of Tdp1 Activity

Tdp1 activity was detected, as described in the work in Reference [39], and consists of fluorescence intensity measurement in a reaction of quencher removal from a fluorophore quencher–coupled DNA oligonucleotide catalyzed by Tdp1. The reaction was carried out at different concentrations of inhibitors (the control samples contained 1% of DMSO). The reaction mixtures contained Tdp1 buffer (50 mM Tris-HCl pH 8.0, 50 mM NaCl, and 7 mM β-mercaptoethanol), 50 nM biosensor, and an inhibitor being tested. Purified Tdp1 (1.5 nM) triggered the reaction. The biosensor (5′-[FAM] AAC GTC AGGGTC TTC C [BHQ]-3′) was synthesized in the Laboratory of Biomedical Chemistry at the Institute of Chemical Biology and Fundamental Medicine (Novosibirsk, Russia).

The reactions were incubated on a POLARstar OPTIMA fluorimeter (BMG LABTECH, GmbH, Ortenberg, Germany) to measure fluorescence every 55 s (ex. 485/em. 520 nm) during the linear phase (here, data from minute 0 to minute 8). The values of IC_50_ were determined using a six-point concentration response curve in a minimum of three independent experiments and were calculated using MARS Data Analysis 2.0 (BMG LABTECH, Ortenberg, Germany).

#### 3.2.2. Cytotoxicity Assays

Cytotoxicity of the compounds to HeLa (human cervical cancer) cell line was examined using the EZ4U Cell Proliferation and Cytotoxicity Assay (Biomedica, Vienna, Austria), according to the manufacturer’s protocols. The cells were grown in Iscove’s modified Dulbecco’s medium (IMDM) with 40 μg/mL gentamicin, 50 IU/mL penicillin, 50 μg/mL streptomycin (MP Biomedicals, Waltham, MA, USA), and 10% of fetal bovine serum (Biolot, Saint Petersburg, Russia) in a 5% CO2 atmosphere. After the formation of a 30–50%-monolayer, the tested compounds were added to the medium. The volume of the added reagents was 1/100 of the total volume of the culture medium, and the amount of DMSO was 1% of the final volume. Control cells were grown in the presence of 1% DMSO. The cell culture was monitored for 3 days.

### 3.3. Molecular Modeling and Screening

The compounds were docked against the crystal structure of Tdp1 (PDB ID: 6DIE, resolution 1.78 Å) [41], which was obtained from the Protein Data Bank (PDB) [52,53]. The Scigress version FJ 2.6 program [54] was used to prepare the crystal structure for docking, i.e., the hydrogen atoms were added, the co-crystallized ligand benzene-1,2,4-tricarboxylic acid was removed as well as crystallographic water molecules, except HOH 814, 821 and 1078. The waters were set on toggle-bound or displaced by the ligand during docking, and spin-automatic optimization of the orientation of the hydrogen atoms. The Scigress software suite was also used to build the inhibitors and the MM2 [55] force field was applied to identify the global minimum using the CONFLEX method [56], followed by structural optimization, as well as running the 10 ps molecular dynamics (MD) run at 1000 K. The docking center was defined as the position of a carbon on the ring of the co-crystallized benzene-1,2,4-tricarboxylic acid (x = −6.052, y = −14.428, z = 33.998) with 10 Å radius. Fifty docking runs were allowed for each ligand with default search efficiency (100%). The basic amino acids lysine and arginine were defined as protonated. Furthermore, aspartic and glutamic acids were assumed to be deprotonated. The GoldScore (GS) [57] and ChemScore (CS) [58,59] ChemPLP (Piecewise Linear Potential) [60] and ASP (Astex Statistical Potential) [61] scoring functions were implemented to predict the binding modes and relative energies of the ligands using the GOLD v5.4.1 software suite.

The QikProp 6.2 [62] software package was used to calculate the molecular descriptors of the molecules. The reliability of it is QikProp, which is established for the calculated descriptors [63]. The Known Drug Indexes (KDI) were calculated from the molecular descriptors as described by Eurtivong and Reynisson [50]. For application in Excel, columns for each property were created and the following equations used to derive the KDI numbers for each descriptor: KDI MW: = EXP(−((MW − 371.76)^2^)/((2 × 112.76)^2^)), KDI Log P: = EXP(−((LogP − 2.82)^2^)/((2 × 2.21)^2^)), KDI HD: = EXP(−((HD − 1.88)^2^)/((2 × 1.7)^2^)), KDI HA: = EXP(−((HA − 5.72)^2^)/((2 × 2.86)^2^)), KDI RB = EXP(−((RB − 4.44)^2^)/((2 × 3.55)^2^)), and KDI PSA: =EXP(−((PSA − 79.4)^2^)/((2 × 54.16)^2^)). These equations could simply be copied into Excel and the descriptor name (e.g., MW) substituted with the value in the relevant column. In order to derive KDI_2A_, this equation was used: = (KDI MW + KDI LogP + KDI HD + KDI HA + KDI RB + KDI PSA) and for KDI_2B_ = (KDI MW × KDI LogP × KDI HD × KDI HA × KDI RB × KDI PSA).

## 4. Conclusions

A new type of berberine derivatives with a fused sultone ring has been discovered. Compounds were obtained in the reaction of berberrubine with sulphochlorides, as well as with classical sulphonates. Investigation of the reaction mechanisms suggests pathways for the formation of sultones, including both a stepwise and synchronous addition of sulphochloride with the latter in the main pathway. Based on this observation, conditions for the selective formation of sultones in good yields were developed. We showed that brominated analogues of berberrubine reacted with sulphochlorides similarly to berberrubine with the formation of 12-Br-sultones. All the synthesized compounds were tested for their inhibitory activity against Tdp1, a promising target for antitumor therapie. It was shown that these compounds, unlike their non-cyclic counterparts (not exhibiting activity against Tdp1) are active against Tdp1 at micromolar and submicromolar concentrations, and the introduction of Br to the 12 position contributes to an increase in the targeting activity. Compound **18c** can be considered to be the lead compound, since it most effectively inhibits Tdp1, is non-toxic, and enhances the cytotoxic effect of topotecan on HeLa cells. The modelling revealed two main binding modes of the ligands, both involving the 1,4-butanesultone ring system. The bromine substituent is not predicted to form direct bonds with Tdp1, rather it is pointing into the water environment and contributes to the binding by aiding the entropic push of the brominated ligands into the binding pocket.

## Figures and Tables

**Table 1:** Tdp1 inhibiting activity (IC_50_) of berberrubine sultones and their 12-brominated analogs.

	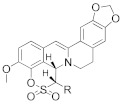			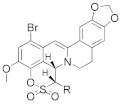		
	Code	IC_50_, µM	CC_50_, µM HeLa	Code	IC_50_, µM	CC_50_, µM HeLa
R = H	**10a**	1.53 ± 0.18	>100	**18a**	0.92 ± 0.05	>100
R = Me	**10b**	2.13 ± 0.16	>100	**18b**	1.13 ± 0.20	>100
R = Et	**10c**	5.50 ± 0.10	>100	**18c**	0.56 ± 0.02	>100
R = Pr	**10d**	1.12 ± 0.18	>100	**18d**	0.78 ± 0.03	>100

## Data Availability

The data presented in this study are available on request from the corresponding author.

## Supplementary Materials

The following are available online, NMR ^1^H and ^13^C spectra of the compounds 10a–10d, 16a, 18a–18d, 2D NMR (COSY and HSQC) spectra of the compound 10b, Table S1: The binding affinities as predicted by the scoring functions used, Table S2: The molecular descriptors and their corresponding Known Drug Indexes 2a and 2b (KDI2a/2b), Table S3: Definition of lead-like, drug-like and Known Drug Space (KDS) in terms of molecular descriptors. The values given are the maxima for each descriptor for the volumes of chemical space used.

## Sample Availability

Samples of the compounds presented in this study are available on request from the authors.

## Abbreviations

Tdp1	Tyrosyl-DNA phosphodiesterase 1
DMSO	Dimethyl sulfoxide

## References

[1] Grycová L., Dostá J., Marek R. (2007). Quaternary protoberberine alkaloids. Phytochemistry.

[2] Wang Y., Zidichouski J.A. (2018). Update on the Benefits and Mechanisms of Action ofthe Bioactive Vegetal Alkaloid Berberine on Lipid Metabolism and Homeostasis. Cholesterol.

[3] Li D.-D., Yu P., Xiao W., Wang Z.-Z., Zhao L.-G. (2020). Berberine: A Promising Natural Isoquinoline Alkaloid for Development of Hypolipidemic Drug. Curr. Top. Med. Chem..

[4] Cicero A.F.G., Tartagni E. (2012). Antidiabetic Properties of Berberine: From Cellular Pharmacology to Clinical Effects. Hosp. Pract..

[5] Vuddanda P.R., Chakraborty S., Singh S. (2010). Berberine: A potential phytochemical with multispectrum therapeutic activities. Expert Opin. Investig. Drugs.

[6] Singh I.P., Mahajan S. (2013). Berberine and its derivatives: A patent review (2009–2012). Expert Opin. Ther. Pat..

[7] Yao L., Wu L.-L., Li Q., Hu Q.-M., Zhang S.-Y., Liu K., Jiang J.-Q. (2018). Novel berberine derivatives: Design, synthesis, antimicrobial effects, and molecular docking studies. Chin. J. Nat. Med..

[8] Park K.D., Lee J.H., Kim S.H., Kang T.H., Moon J.S., Kim S.U. (2006). Synthesis of 13-(substituted benzyl) berberine and berberrubine derivatives as antifungal agents. Bioorganic Med. Chem. Lett..

[9] Nechepurenko I.V., Boyarskikh U.A., Khvostov M.V., Baev D.S., Komarova N.I., Filipenko M.L., Tolstikova T.G., Salakhutdinov N.F. (2015). Hypolipidemic Berberine Derivatives with a Reduced Aromatic Ring C. Chem. Nat. Compd..

[10] Zhang C., Sheng J., Li G., Zhao L., Wang Y., Yang W., Yao X., Sun L., Zhang Z., Cui R. (2019). Effects of Berberine and Its Derivatives on Cancer: A Systems Pharmacology Review. Front. Pharm..

[11] Liu H., Li G., Wang J., Liu J. (2011). Compounds and Compositions for Modulating Lipid Levels and Methods of Preparing Same. U.S. Patent Application No..

[12] Wang Y.-X., Liu L., Zeng Q.-X., Fan T.-Y., Jiang J.-D., Deng H.-B., Song D.-Q. (2017). Synthesis and Identification of Novel Berberine Derivatives as Potent Inhibitors against TNF-α-Induced NF-κB Activation. Molecules.

[13] Kawale A.S., Povirk L.F. (2018). Tyrosyl-DNA phosphodiesterases: Rescuing the genome from the risks of relaxation. Nucleic Acids Res..

[14] Cuya S.M., Comeaux E.Q., Wanzeck K., Yoon K.J., van Waardenburg R.C. (2016). Dysregulated human Tyrosyl-DNA phosphodiesterase I acts as cellular toxin. Oncotarget.

[15] Katyal S., El-Khamisy S.F., Russell H.R., Li Y., Ju L., Caldecott K.W., McKinnon P.J. (2007). Tdp1 facilitates chromosomal single-strand break repair in neurons and is neuroprotective in vivo. EMBO J..

[16] Alagoz M., Wells O.S., El-Khamisy S.F. (2014). Tdp1 deficiency sensitizes human cells to base damage via distinct topoisomerase I and PARP mechanisms with potential applications for cancer therapy. Nucleic Acids Res..

[17] Huang H.C., Liu J., Baglo Y., Rizvi I., Anbil S., Pigula M., Hasan T. (2018). Mechanism-informed Repurposing of Minocycline Overcomes Resistance to Topoisomerase Inhibition for Peritoneal Carcinomatosis. Mol. Cancer.

[18] Nivens M.C., Felder T., Galloway A.H., Pena M.M., Pouliot J.J., Spencer H.T. (2004). Engineered resistance to camptothecin and antifolates by retroviral coexpression of tyrosyl DNA phosphodiesterase-I and thymidylate synthase. Cancer Chemother. Pharm..

[19] Barthelmes H.U., Habermeyer M., Christensen M.O., Mielke C., Interthal H., Pouliot J.J., Boege F., Marko D. (2004). Tdp1 overexpression in human cells counteracts DNA damage mediated by topoisomerases I and II. J. Biol. Chem..

[20] Meisenberg C., Gilbert D.C., Chalmers A., Haley V., Gollins S., Ward S.E., El-Khamisy S.F. (2015). Clinical and cellular roles for Tdp1 and Top1 in modulating colorectal cancer response to irinotecan. Mol. Cancer.

[21] Khomenko T., Zakharenko A., Odarchenko T., Arabshahi H.J., Sannikova V., Zakharova O., Korchagina D., Reynisson J., Volcho K., Salakhutdinov N. (2016). New Inhibitors of Tyrosyl-DNA Phosphodiesterase I (Tdp 1) Combining 7-Hydroxycoumarin and Mono-terpenoid Moieties. Bioorg. Med. Chem..

[22] Ponomarev K.Y., Suslov E.V., Zakharenko A.L., Zakharova O.D., Rogachev A.D., Korchagina D.V., Zafar A., Reynisson J., Nefedov A.A., Volcho K.P. (2018). Aminoadamantanes Containing Monoterpene-derived Fragments as Potent Tyrosyl-DNA phosphodiesterase 1 Inhibitors. Bioorganic Chem..

[23] Zakharova O., Luzina O., Zakharenko A., Sokolov D., Filimonov A., Dyrkheeva N., Chepanova A., Ilina E., Ilyina A., Klabenkova K. (2018). Synthesis and evaluation of aryliden- and hetarylidenfuranone derivatives of usnic acid as highly potent Tdp1 inhibitors. Bioorganic Med. Chem..

[24] Mozhaitsev E.S., Zakharenko A.L., Suslov E.V., Korchagina D.V., Zakharova O.D., Vasil’eva I.A., Chepanova A.A., Black E., Patel J., Chand R. (2019). Novel Inhibitors of DNA Repair Enzyme Tdp1 Combining Monoterpenoid and Adamantane Fragments. Anti Cancer Agents Med. Chem..

[25] Filimonov A.S., Chepanova A.A., Luzina O.A., Zakharenko A.L., Zakharova O.D., Ilina E.S., Dyrkheeva N.S., Kuprushkin M.S., Kolotaev A.V., Khachatryan D.S. (2019). New Hydrazinothiazole Derivatives of Usnic Acid as Potent Tdp1 Inhibitors. Molecules.

[26] Il’ina I.V., Dyrkheeva N.S., Zakharenko A.L., Sidorenko A.Y., Li-Zhulanov N.S., Korchagina D.V., Chand R., Ayine-Tora D.M., Chepanova A.A., Zakharova O.D. (2020). Design, Synthesis, and Biological Investigation of Novel Classes of 3-Carene-Derived Potent Inhibitors of Tdp1. Molecules.

[27] Luzina O., Filimonov A., Zakharenko A., Chepanova A., Zakharova O., Ilina E., Dyrkheeva N., Likhatskaya G., Salakhutdinov N., Lavrik O. (2020). Usnic Acid Conjugates with Monoterpenoids as Potent Tyrosyl-DNA Phosphodiesterase 1 Inhibitors. J. Nat. Prod..

[28] Zakharenko A.L., Luzina O.A., Sokolov D.N., Zakharova O.D., Rakhmanova M.E., Chepanova A.A., Dyrkheeva N.S., Lavrik O.I., Salakhutdinov N.F. (2017). Usnic Acid Derivatives Are Effective Inhibitorsof Tyrosyl-DNA Phosphodiesterase 1. Russ. J. Bioorg. Chem..

[29] Zakharenko A.L., Luzina O.A., Sokolov D.N., Kaledin V.I., Nikolin V.P., Popova N.A., Patel J., Zakharova O.D., Chepanova A.A., Zafar A. (2019). Novel tyrosyl-DNA phosphodiesterase 1 in-hibitors enhance the therapeutic impact of topotecan on in vivo tumor models. Eur. J. Med. Chem..

[30] Khomenko T.M., Zakharenko A.L., Chepanova A.A., Ilina E.S., Zakharova O.D., Kaledin V.I., Nikolin V.P., Popova N.A., Korchagina D.V., Reynisson J. (2020). New Promising Inhibitors of Tyrosyl-DNA Phosphodiesterase I (Tdp 1) Combining 4-Arylcoumarin and Monoterpenoid Moieties as Components of Complex Antitumor Therapy. Int. J. Mol. Sci..

[31] Laev S.S., Salakhutdinov N.F., Lavrik O.I. (2016). Tyrosyl-DNA phosphodiesterase inhibitors: Progress and potential. Bioorg. Med. Chem..

[32] Huang S.N., Pommier Y., Marchand C. (2011). Tyrosyl-DNA Phosphodiesterase 1 (Tdp1) inhibitors. Expert Opin. Ther. Pat..

[33] Salomatina O.V., Popadyuk I.I., Zakharenko A.L., Zakharova O.D., Fadeev D.S., Komarova N.I., Reynisson J., Arabshahi H.I., Chand R., Volcho K.P. (2018). Novel Semisynthetic Derivatives of Bile Acids as Effective Tyrosyl-DNA Phosphodiesterase 1 Inhibitors. Molecules.

[34] Zakharenko A., Luzina O., Koval O., Nilov D., Gushchina I., Dyrkheeva N., Švedas V., Salakhutdinov N., Lavrik O. (2016). Tyrosyl-DNA Phosphodiesterase 1 Inhibitors: Usnic Acid Enamines Enhance the Cytotoxic Effect of Camptothecin. J. Nat. Prod..

[35] Chepanova A.A., Li-Zhulanov N.S., Sukhikh A.S., Zafar A., Reynisson J., Zakharenko A.L., Zakharova O.D., Korchagina D.V., Volcho K.P., Salakhutdinov N.F. (2019). Effective Inhibitors of Tyrosy l-DNA Phosphodiesterase 1 Based on Monoterpenoids as Potential Agents for Antitumor Therapy. Russ. J. Bioorg. Chem..

[36] Zhang X.-R., Wang H.-W., Tang W.-L., Zhang Y., Yang H., Hu D.-X., Ravji A., Marchand C., Kiselev E., Ofori-Atta K. (2018). Discovery, Synthesis, and Evaluation of Oxynitidine Derivatives as Dual Inhibitors of DNA Topoisomerase IB (TOP1) and Tyrosyl-DNA Phosphodiesterase 1 (TDP1), and Potential Antitumor Agents. J. Med. Chem..

[37] Habtemariam S. (2020). Recent Advances in Berberine Inspired AnticancerApproaches: From Drug Combination to NovelFormulation Technology and Derivatization. Molecules.

[38] Gladkova E.D., Nechepurenko I.V., Bredikhin R.A., Chepanova A.A., Zakharenko A.L., Luzina O.A., Ilina E.S., Dyrkheeva N.S., Mamontova E.M., Anarbaev R.O. (2020). The First Berberine-based Inhibitors of Tyrosyl-DNA Phosphodiesterase 1 (Tdp1), an Important DNA Repair Enzyme. Int. J. Mol. Sci..

[39] Zakharenko A.L., Khomenko T.M., Zhukova S.V., Koval O.A., Zakharova O.D., Anarbaev R.O., Lebedeva N.A., Korchagina D.V., Komarova N.I., Vasiliev V.G. (2015). Synthesis and biological evaluation of novel tyrosyl-DNA phosphodiesterase 1 inhibitors with a benzopentathiepine moiety. Bioorg. Med. Chem..

[40] Chepanova A.A., Mozhaitsev E.S., Munkuev A.A., Suslov E.V., Korchagina D.V., Zakharova O.D., Zakharenko A.L., Patel J., Ayine-Tora D.M., Reynisson J. (2019). The Development of Tyrosyl-DNA Phosphodiesterase 1 Inhibitors. Combination of Monoterpene and Adamantine Moieties via Amide or Thioamide Bridges. Appl. Sci..

[41] Lountos G.T., Zhao X.Z., Kiselev E., Tropea J.E., Needle D., Pommier Y., Burke T.R., Waugh D.S. (2019). Identification of a Ligand Binding Hot Spot and Structural Motifs Replicating Aspects of Tyrosyl-DNA Phosphodiesterase I (Tdp1) Phosphoryl Recognition by Crystallographic Fragment Cocktail Screening. Nucleic Acids Res..

[42] Lee G.E., Lee H.-S., Lee S.D., Kim J.-H., Kim W.-K., Kim Y.-C. (2009). Synthesis and structure–activity relationships of novel, substituted 5,6-dihydrodibenzo[a,g]quinolizinium P2X7 antagonists. Bioorg. Med. Chem. Lett..

[43] Opitz G., Mohl H.R. (1969). Disulfene. Angew. Chem. Intern. Ed..

[44] Nechepurenko I.V., Shirokova E.D., Khvostov M.V., Frolova T.S., Sinitsyna O.I., Maksimov A.M., Bredikhin R.A., Komarova N.I., Fadeev D.S., Luzina O.A. (2019). Synthesis, hypolipidemic and antifungal activity of tetrahydroberberrubine sulfonates. Russ. Chem. Bull..

[45] Raghav D., Ashraf S.M., Mohan L., Rathinasamy K. (2017). Berberine Induces Toxicity in HeLa Cells through Perturbation of Microtubule Polymerization by Binding to Tubulin at a Unique Site. Biochemistry.

[46] Li-Zhulanov N.S., Zakharenko A.L., Chepanova A.A., Patel J., Zafar A., Volcho K.P., Salakhutdinov N.F., Reynisson J., Leung I.K.H., Lavrik O.I. (2018). A Novel Class of Tyrosyl-DNA Phosphodiesterase 1 Inhibitors That Contains the Octahydro-2H-chromen-4-ol Scaffold. Molecules.

[47] Mozhaitsev E., Suslov E., Demidova Y., Korchagina D., Volcho K., Zakharenko A., Vasil’eva I., Kupryushkin M., Chepanova A., Ayine-Tora D.M. (2019). The Development of Tyrosyl-DNA Phosphodyesterase 1 (TDP1) Inhibitors Based on the Amines Combining Aromatic/Heteroaromatic and Monoterpenoid Moieties. Lett. Drug Des. Discov..

[48] Salomatina O.V., Popadyuk I.I., Zakharenko A.L., Zakharova O.D., Chepanova A.A., Dyrkheeva N.S., Komarova N.I., Reynisson J., Anarbaev R.O., Salakhutdinov N.F. (2021). Deoxycholic acid as a molecular scaffold for tyrosyl-DNA phosphodiesterase 1 inhibition: A synthesis, structure–activity relationship and molecular modeling study. Steroids.

[49] Zhu F., Logan G., Reynisson J. (2012). Wine Compounds as a Source for HTS Screening Collections. A Feasibiity Study. Mol. Inf..

[50] Eurtivong C., Reynisson J. (2018). The Development of a Weighted Index to Optimise Compound Libraries for High Throughput Screening. Mol. Inf..

[51] Nechepurenko I.V., Komarova N.I., Vasilev V.G., Salakhutdinov N.F. (2012). Synthesis of berberine bromide analogs containing tertiary amides of acetic acid in the 9-*O*-position. Chem. Nat. Compd..

[52] Berman H.M., Westbrook J., Feng Z., Gilliland G., Bhat T.N., Weissig H., Shindyalov I.N., Bourne P.E. (2000). The Protein Data Bank. Nucleic Acids Res..

[53] Berman H., Henrick K., Nakamura H. (2003). Announcing the Worldwide Protein Data Bank. Nat. Struct. Biol..

[54] Scigress Ultra V., F.J 2.6. (EU 3.1.7); Fujitsu Limited: 2008–2016.

[55] Allinger N.L. (1977). Conformational Analysis. 130. MM2. A Hydrocarbon Force Field Utilizing V1 and V2 Torsional Terms. J. Am. Chem. Soc..

[56] Gotō H., Ōsawa E. (1993). An Efficient Algorithm for Searching Low-Energy Conformers of Cyclic and Acyclic Molecules. Perkin 2 Int. J. Phys. Org. Chem..

[57] Jones G., Willet P., Glen R.C., Leach A.R., Taylor R. (1997). Development and Validation of a Genetic Algorithm for Flexible Docking. J. Mol. Biol..

[58] Eldridge M.D., Murray C., Auton T.R., Paolini G.V., Mee P.M. (1997). Empirical Scoring Functions: I. the Development of a Fast Empirical Scoring Function to Estimate the Binding Affinity of Ligands in Receptor Complexes. J. Comp. Aid. Mol. Des..

[59] Verdonk M.L., Cole J.C., Hartshorn M.J., Murray C.W., Taylor R.D. (2003). Improved Protein-Ligand Docking using GOLD. Proteins.

[60] Korb O., Stützle T., Exner T.E. (2009). Empirical Scoring Functions for Advanced Protein−Ligand Docking with PLANTS. J. Chem. Inf. Model..

[61] Mooij W.T.M., Verdonk M.L. (2005). General and Targeted Statistical Potentials for Protein–ligand Interactions. Proteins.

[62] (2009). QikProp.

[63] Ioakimidis L., Thoukydidis L., Naeem S., Mirza A., Reynisson J. (2008). Benchmarking the Reliability of QikProp. Correlation between Experimental and Predicted Values. QSAR Comb. Sci..

