# Supplementary material for: Discovery of Novel Sultone Fused Berberine Derivatives as Promising Tdp1 Inhibitors
Source: Molecules. 2021 Mar 30;26(7):1945. doi: 10.3390/molecules26071945 (PMC8037669; doi:10.3390/molecules26071945)
Supplement: Supplementary file 1 [file molecules-26-01945-s001.pdf]

**SUPPLEMENTARY MATERIALS**

# Discovery of novel sultone fused berberine derivatives as promising Tdp1 inhibitors

Elizaveta D. Gladkova, Arina A. Chepanova, Ekaterina S. Ilina, Alexandra L. Zakharenko, Jóhannes Reynisson, Olga A. Luzina, Konstantin P. Volcho, Olga I. Lavrik and Nariman F. Salakhutdinov

NMR $^{1}H$ and $^{13}C$ spectra of the compounds **5a – 5d**, **12a**, **14a – 14d.**

2D NMR (COSY and HSQC) spectra of the compound **5b**.

**Table S1.** The binding affinities as predicted by the scoring functions used.

**Table S2.** The molecular descriptors and their corresponding Known Drug Indexes 2a and 2b ($KDI_{2a/2b}$).

**Table S3.** Definition of lead-like, drug-like and Known Drug Space (KDS) in terms of molecular descriptors. The values given are the maxima for each descriptor for the volumes of chemical space used.

Compound **5a** (**$^{1}$H NMR, solvent - DMSO-d6**)

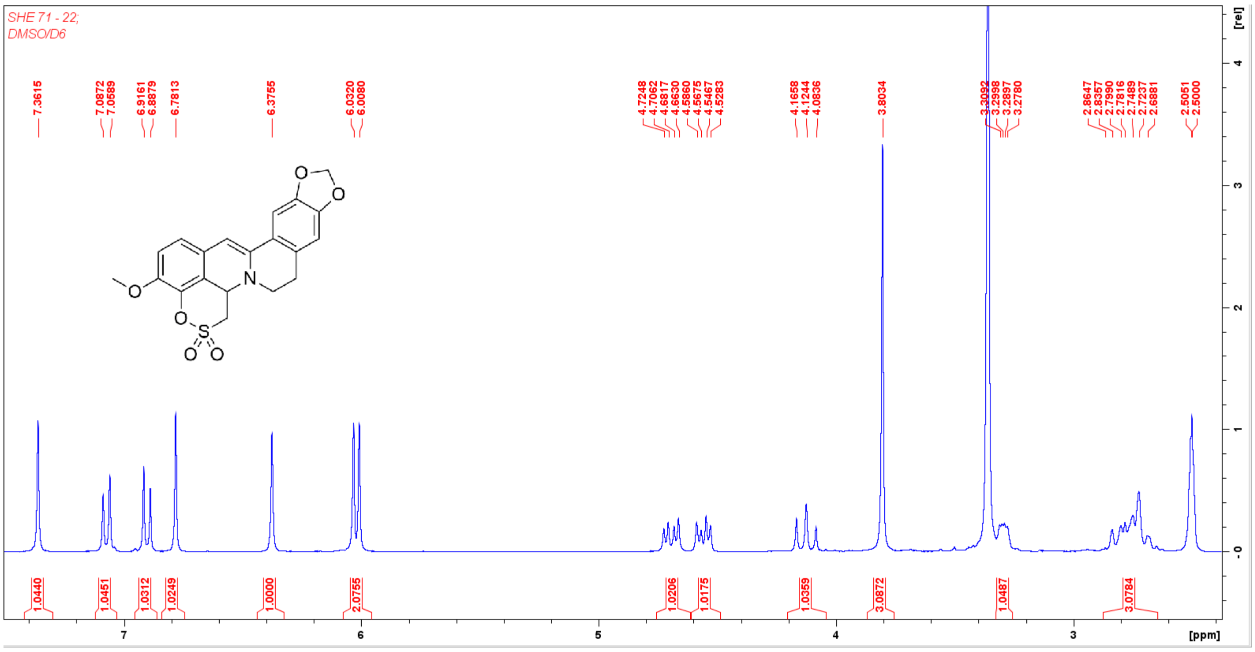


Compound **5a** (**$^{13}$C NMR, solvent – CDCl3**)

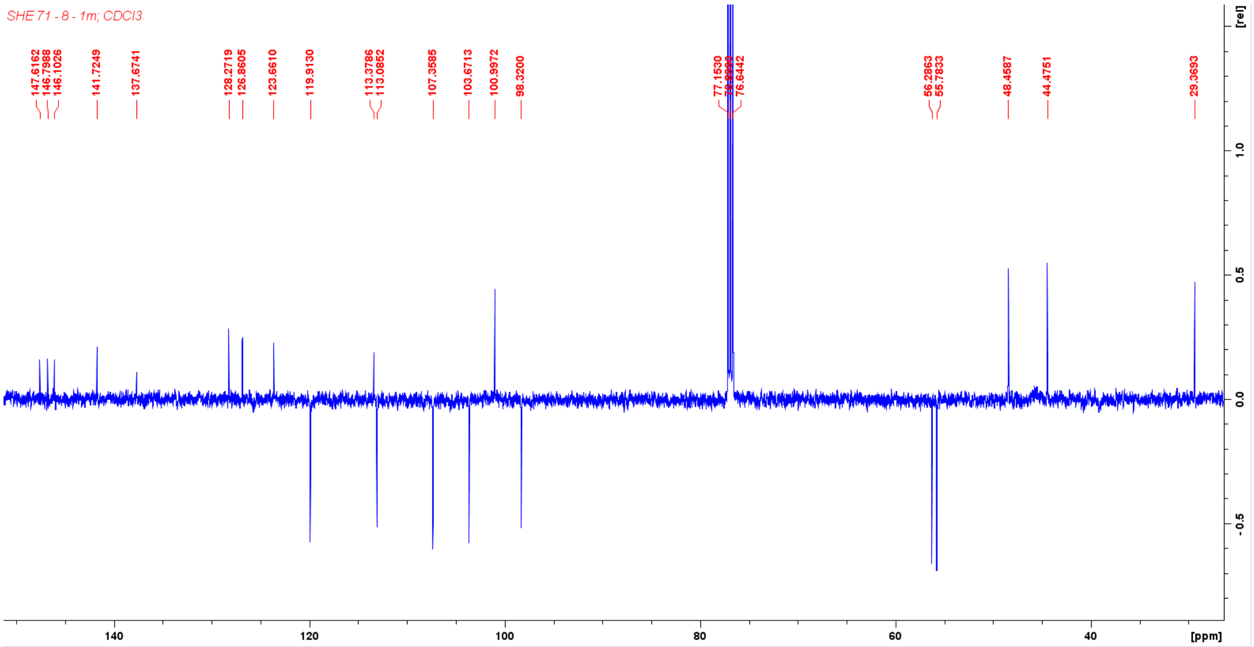

Compound **5b** ($^{1}$H NMR, solvent - DMSO-d6)

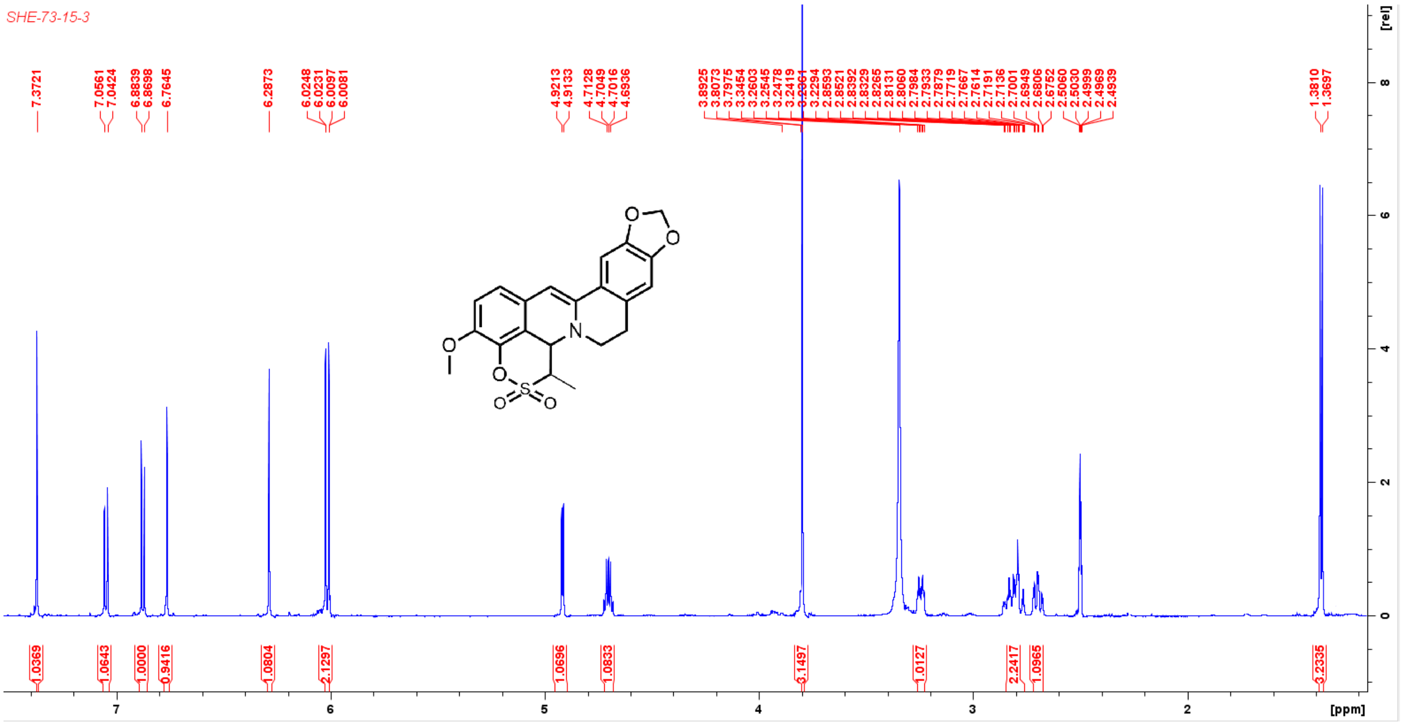

Compound **5b** ($^{13}$C NMR, solvent - DMSO-d6)

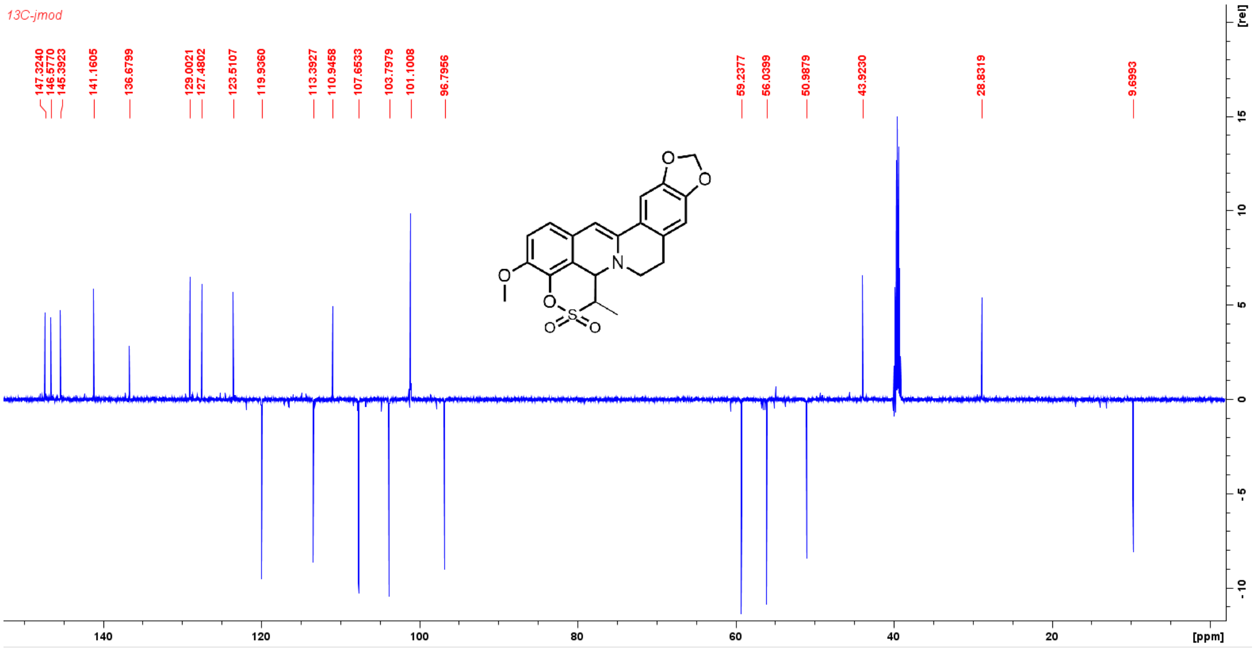

Compound **5c (¹H NMR, solvent - DMSO-d6)**

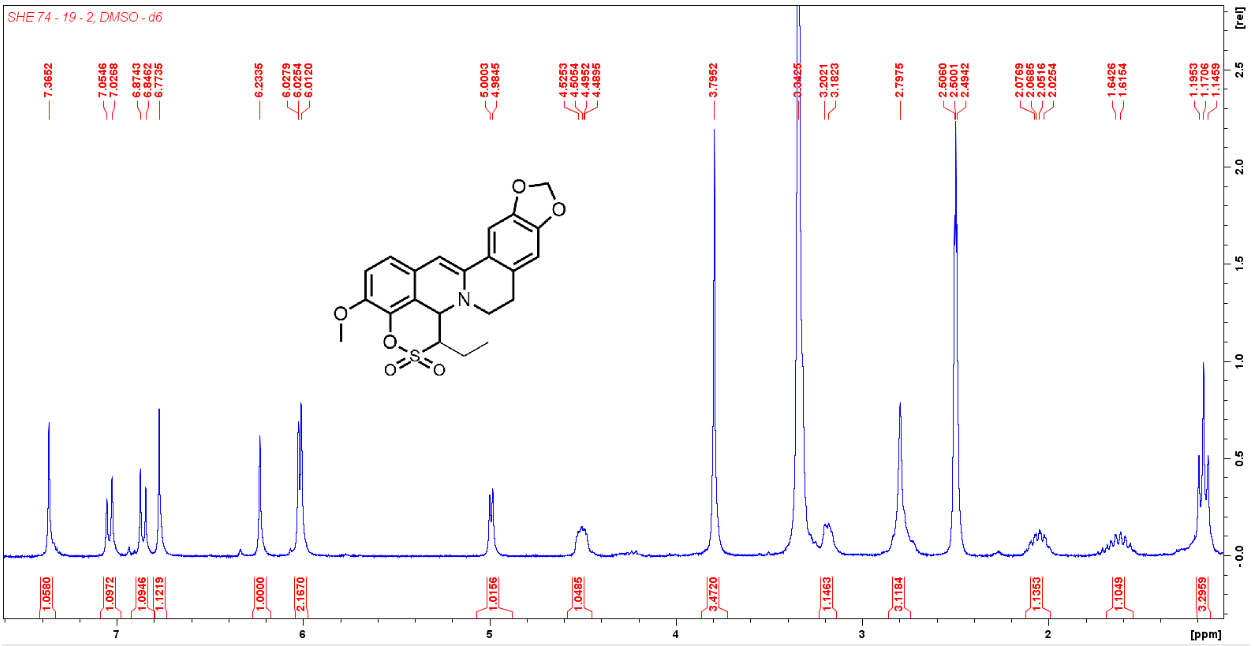


Compound **5c (¹³C NMR, solvent - DMSO-d6)**

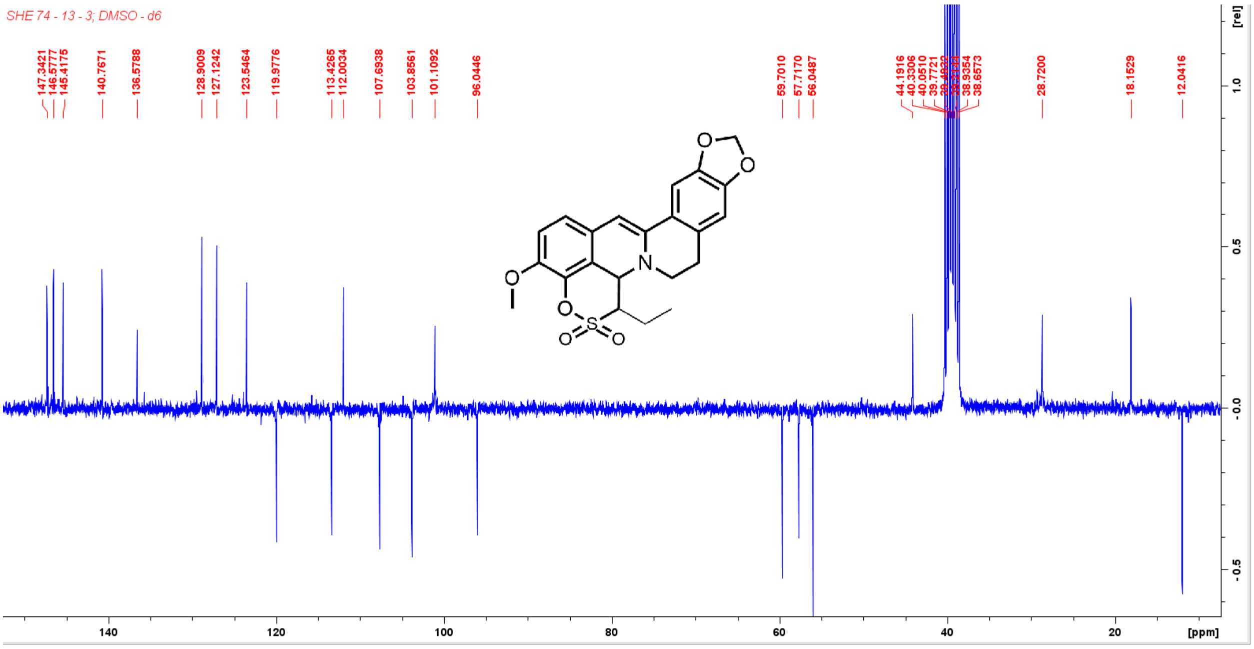

Compound **5d** (**$^{1}$H NMR, solvent - DMSO-d6)**

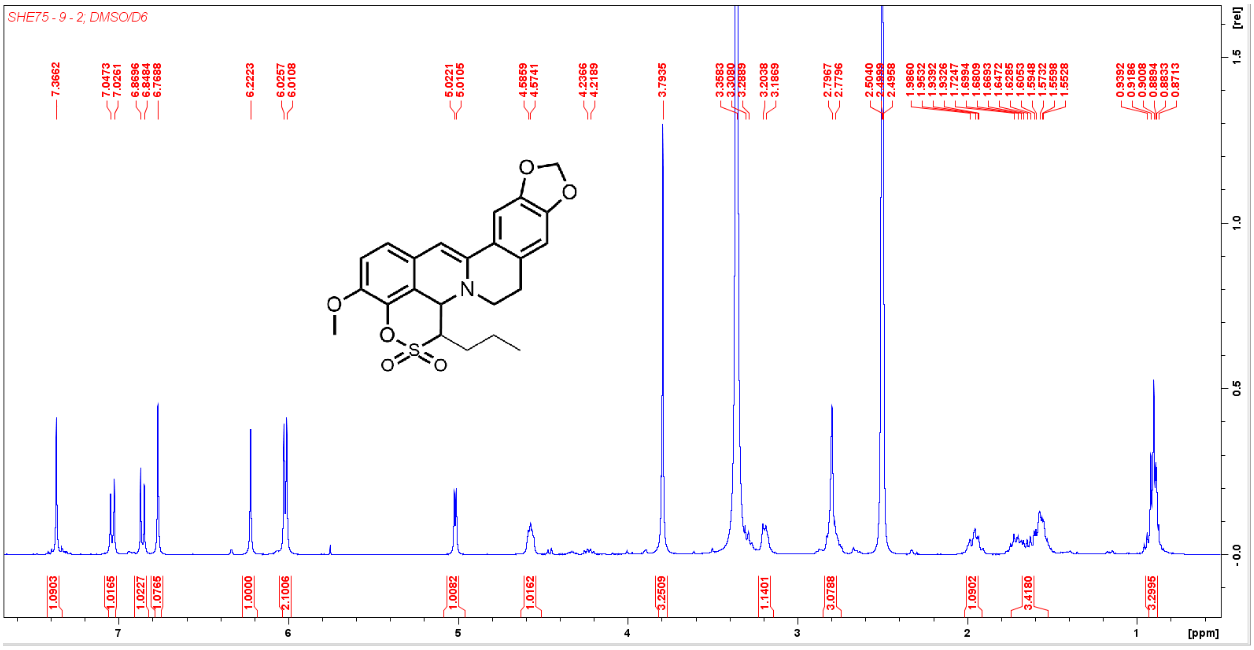


Compound **5d** (**$^{13}$C NMR, solvent - DMSO-d6)**

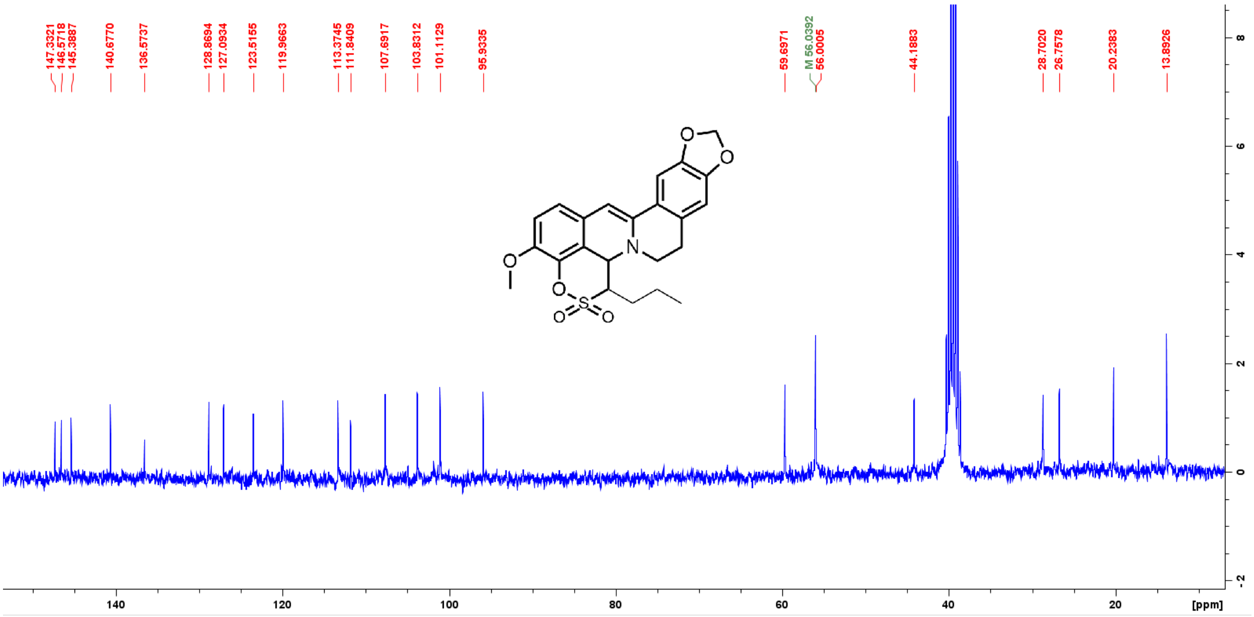

Compound **12a** ($^{1}H$ NMR, solvent - DMSO-d6)

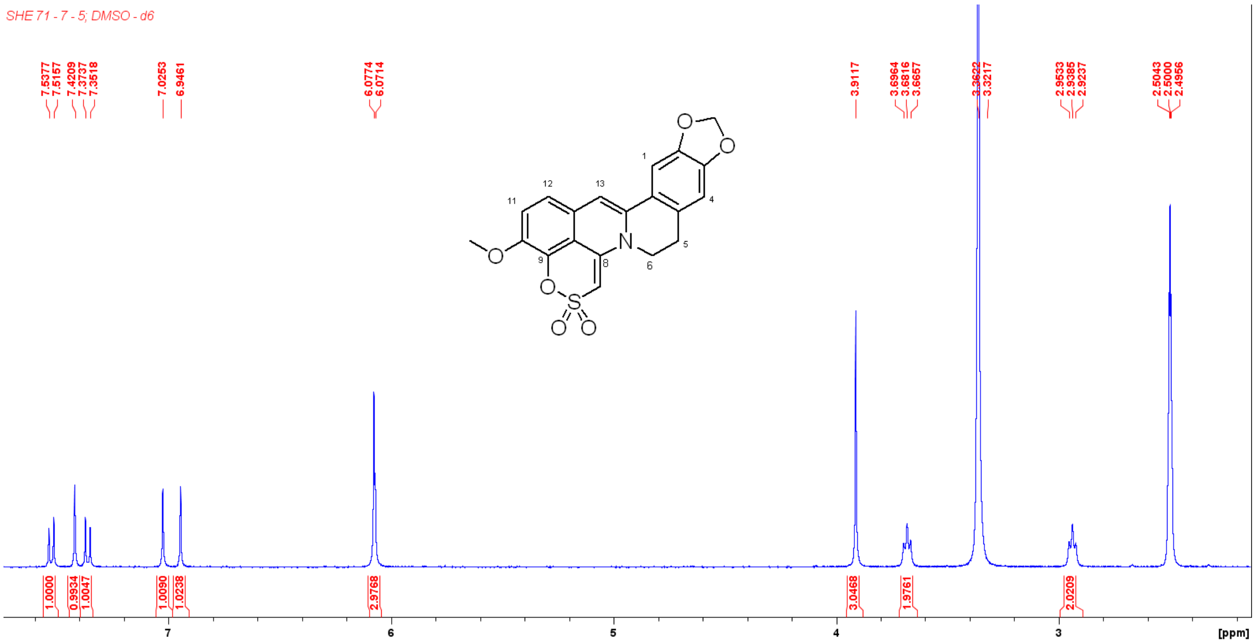

Compound **12a** ($^{13}C$ NMR, solvent - DMSO-d6)

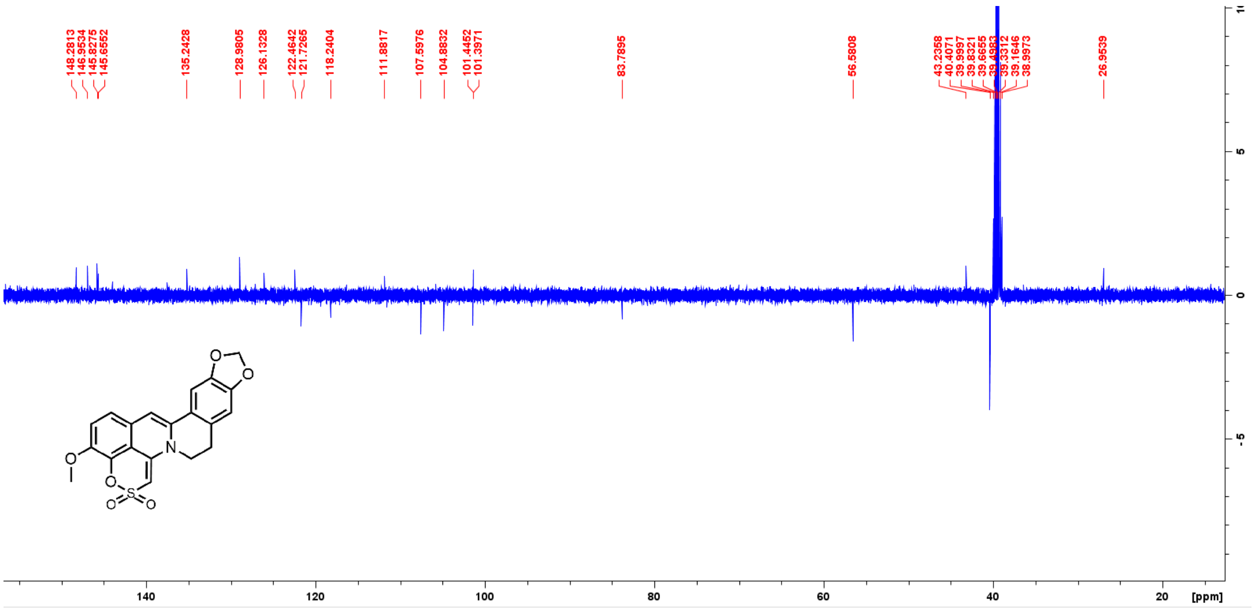

Compound **14a** ($^1$H NMR, solvent - DMSO-d6)

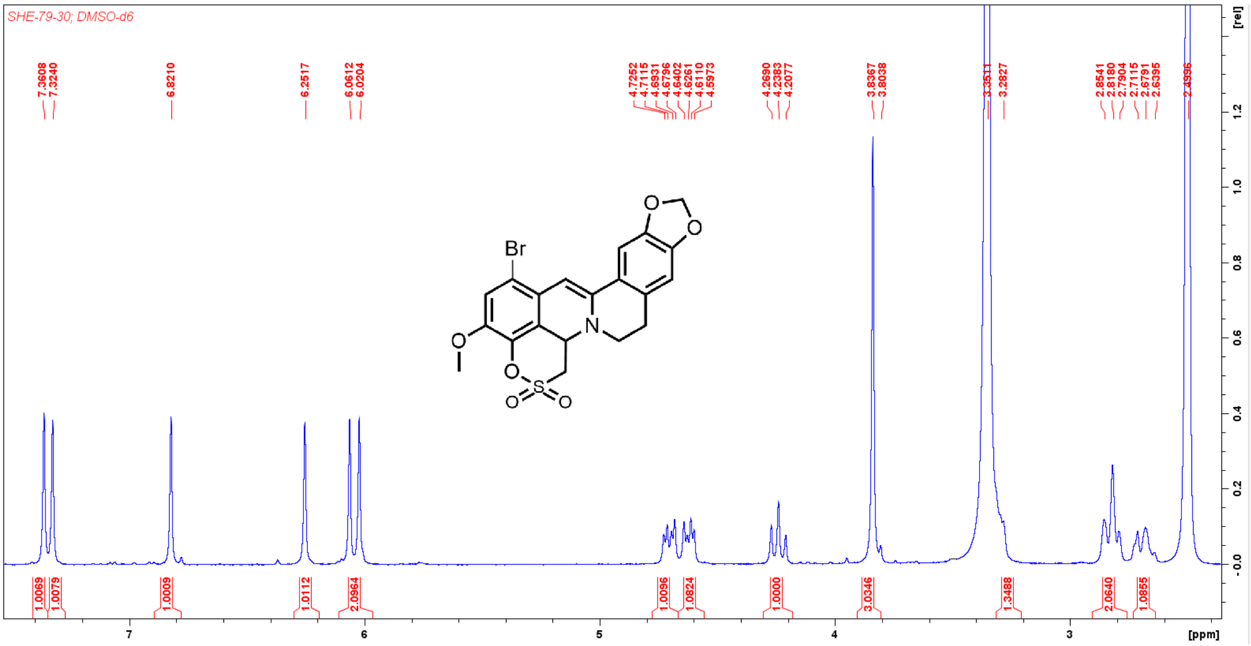


Compound **14a** ($^{13}$C NMR, solvent - DMSO-d6)

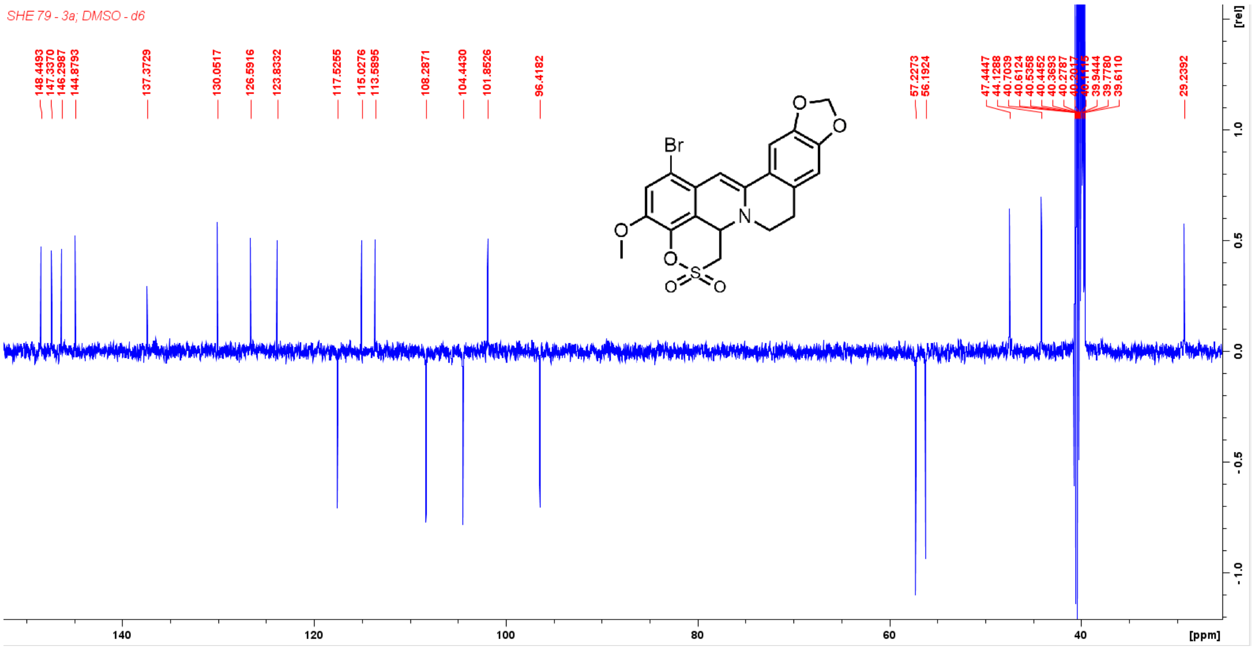

Compound **14b** ($^{1}$H NMR, solvent - DMSO-d6)

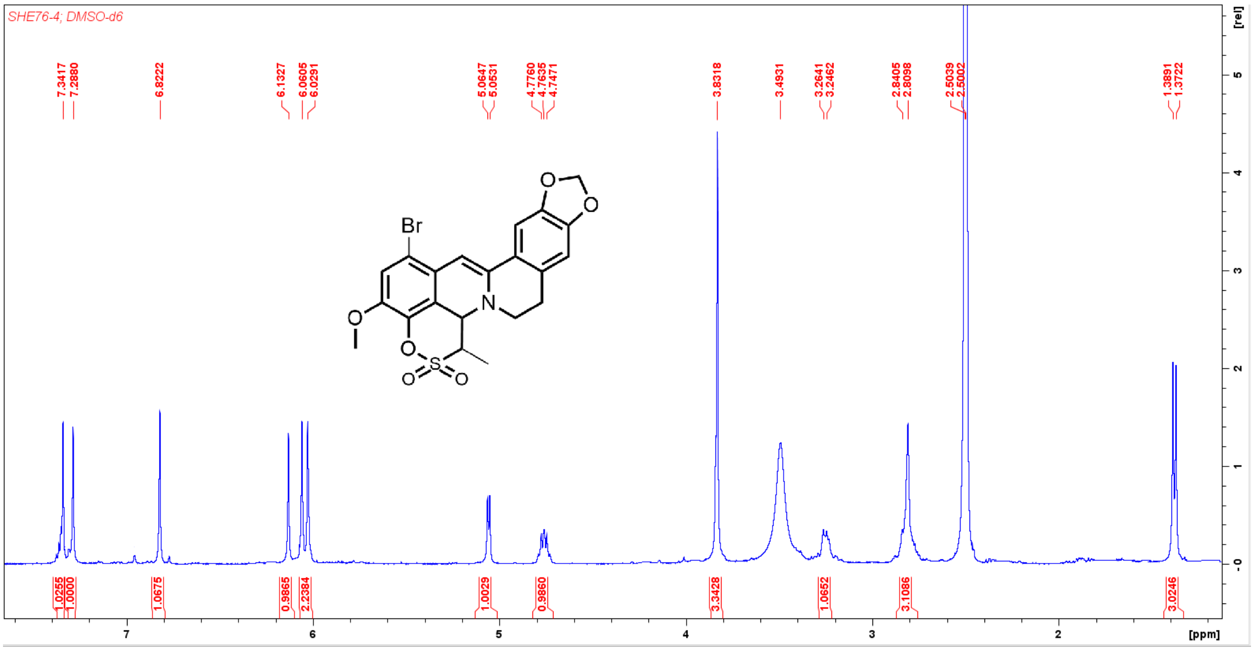


Compound **14b** ($^{13}$C NMR, solvent - DMSO-d6)

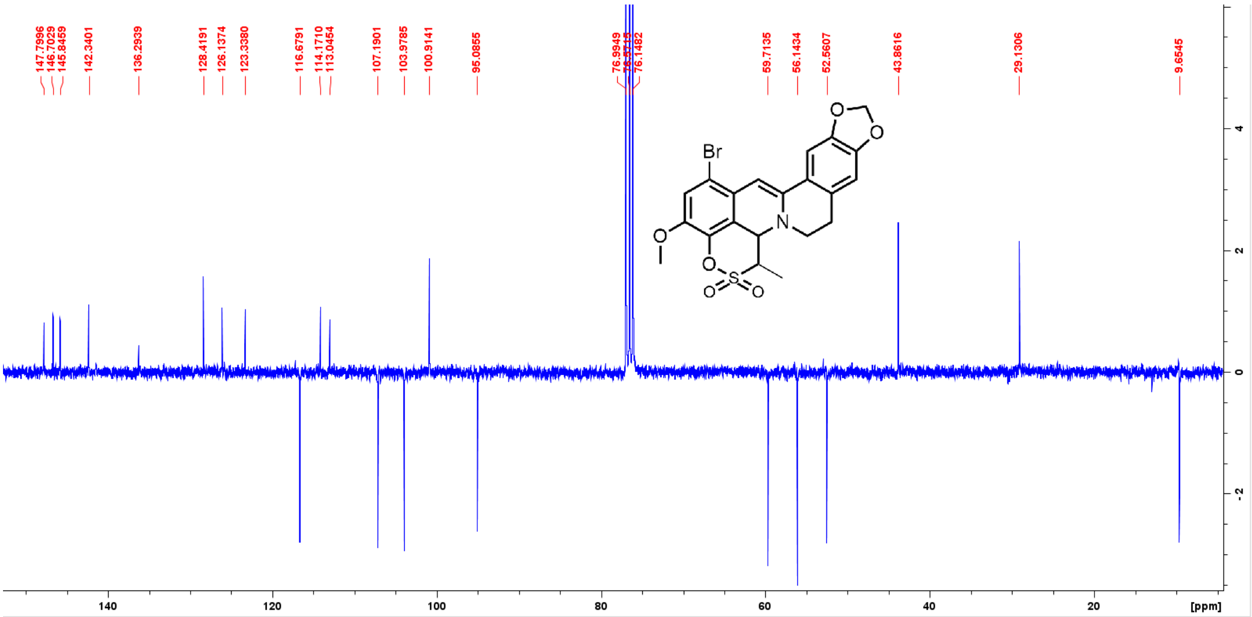

Compound **14c** ($^{1}$H NMR, solvent - DMSO-d6)

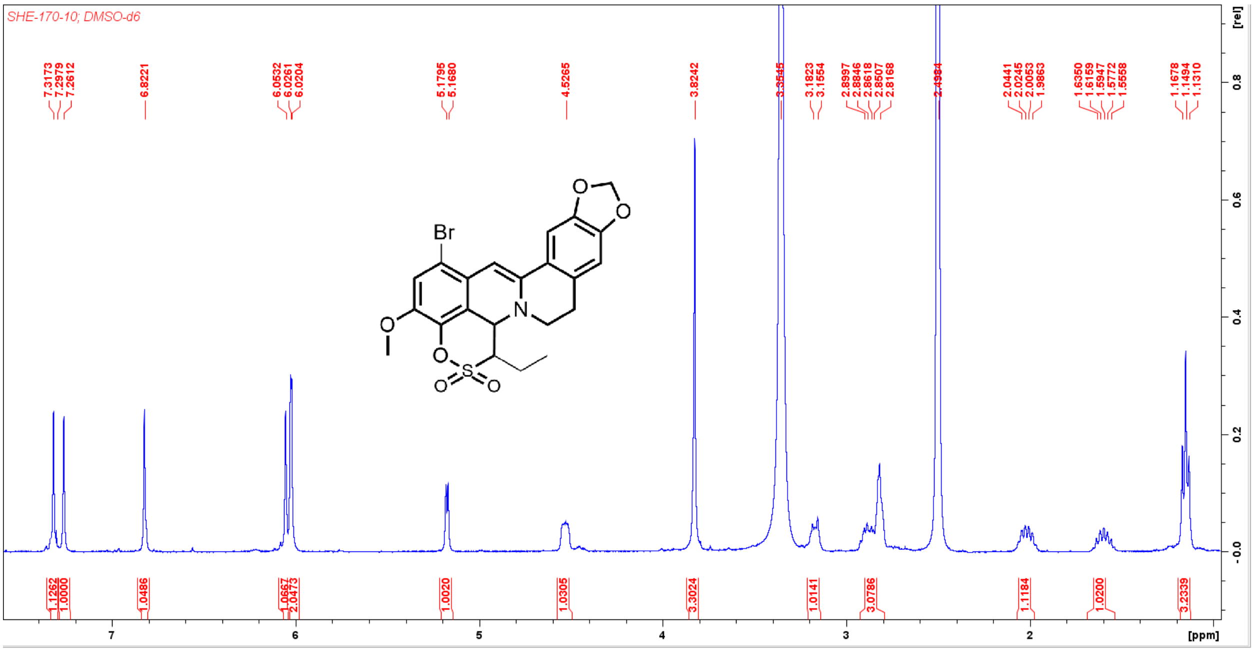


Compound **14c** ($^{13}$C NMR, solvent - DMSO-d6)

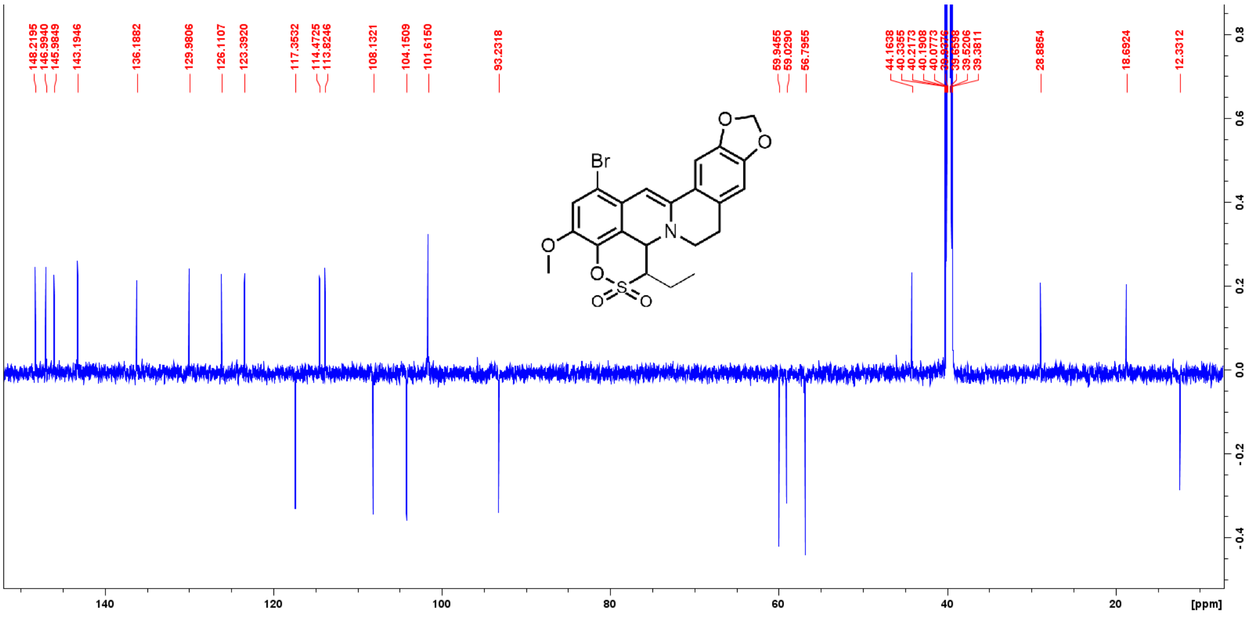

Compound **14d (¹H NMR, solvent - DMSO-d6)**

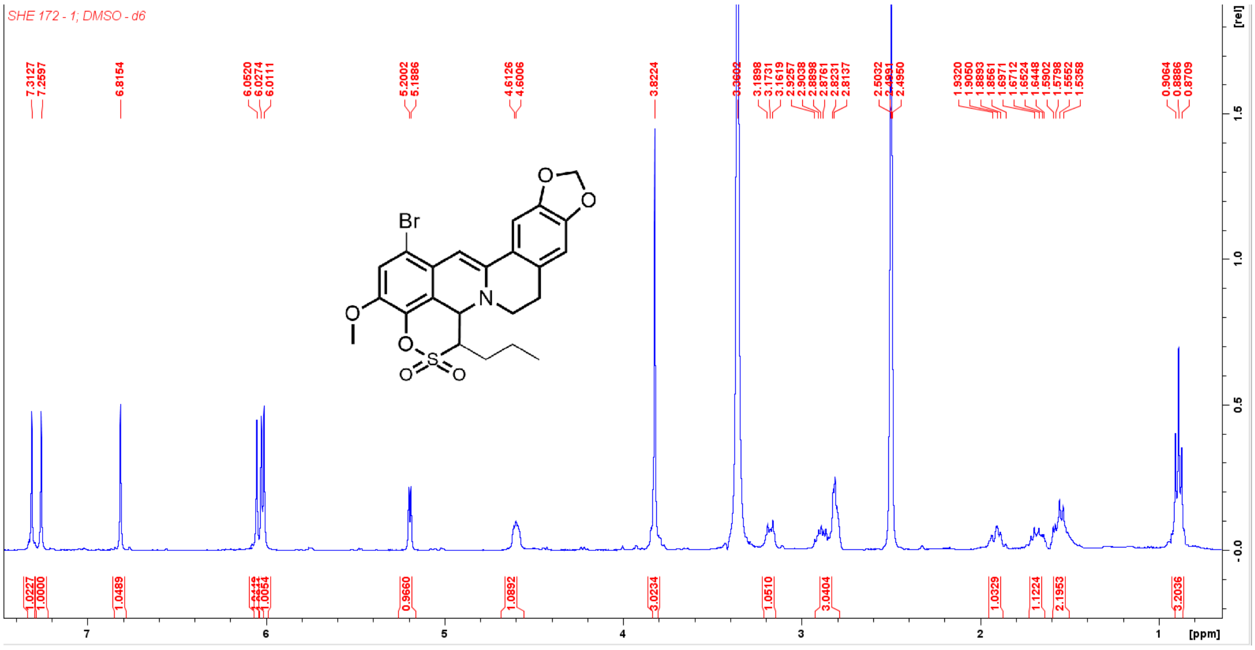

Compound **14d (¹³C NMR, solvent - DMSO-d6)**

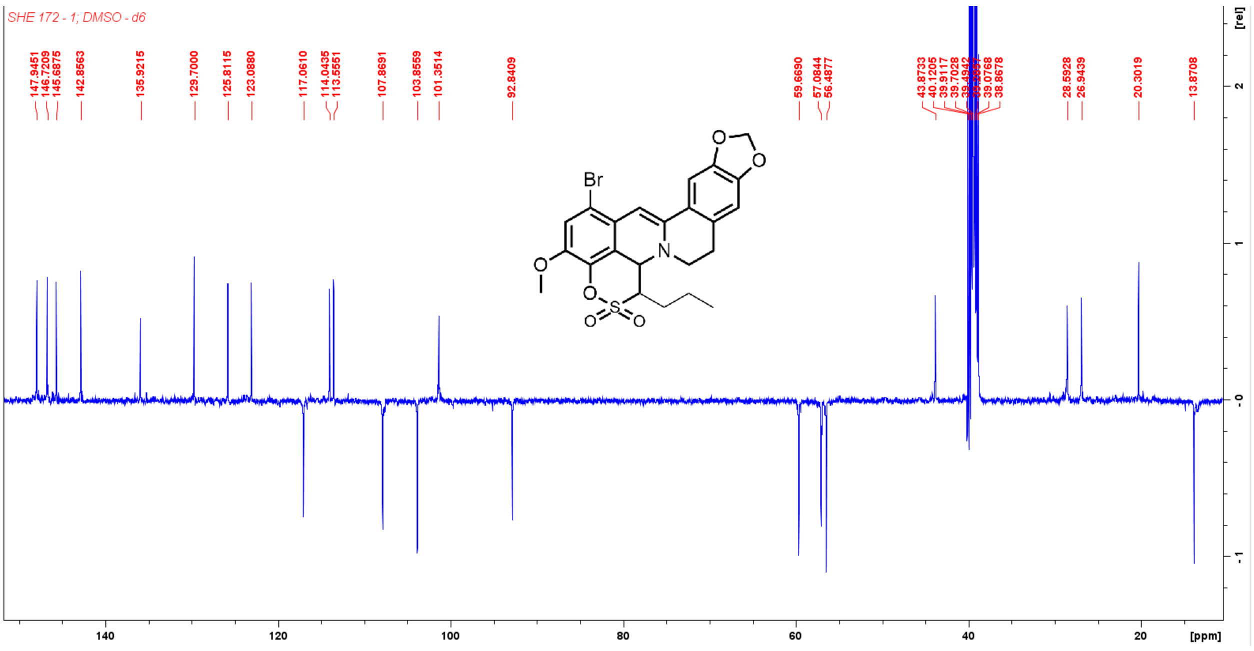

Compound **5b (COSY 2D NMR, solvent - DMSO-d6)**

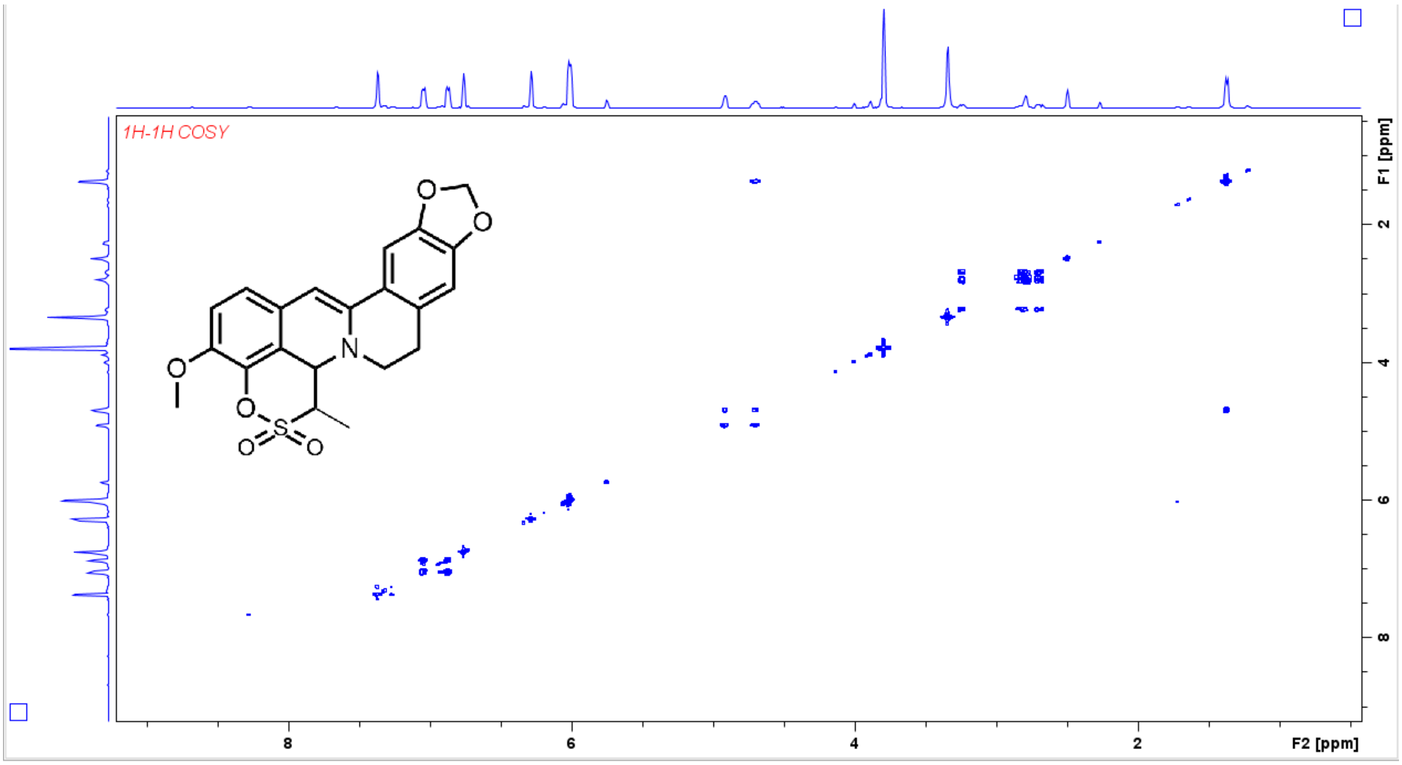


Compound **5b (HSQC 2D NMR, solvent - DMSO-d6)**

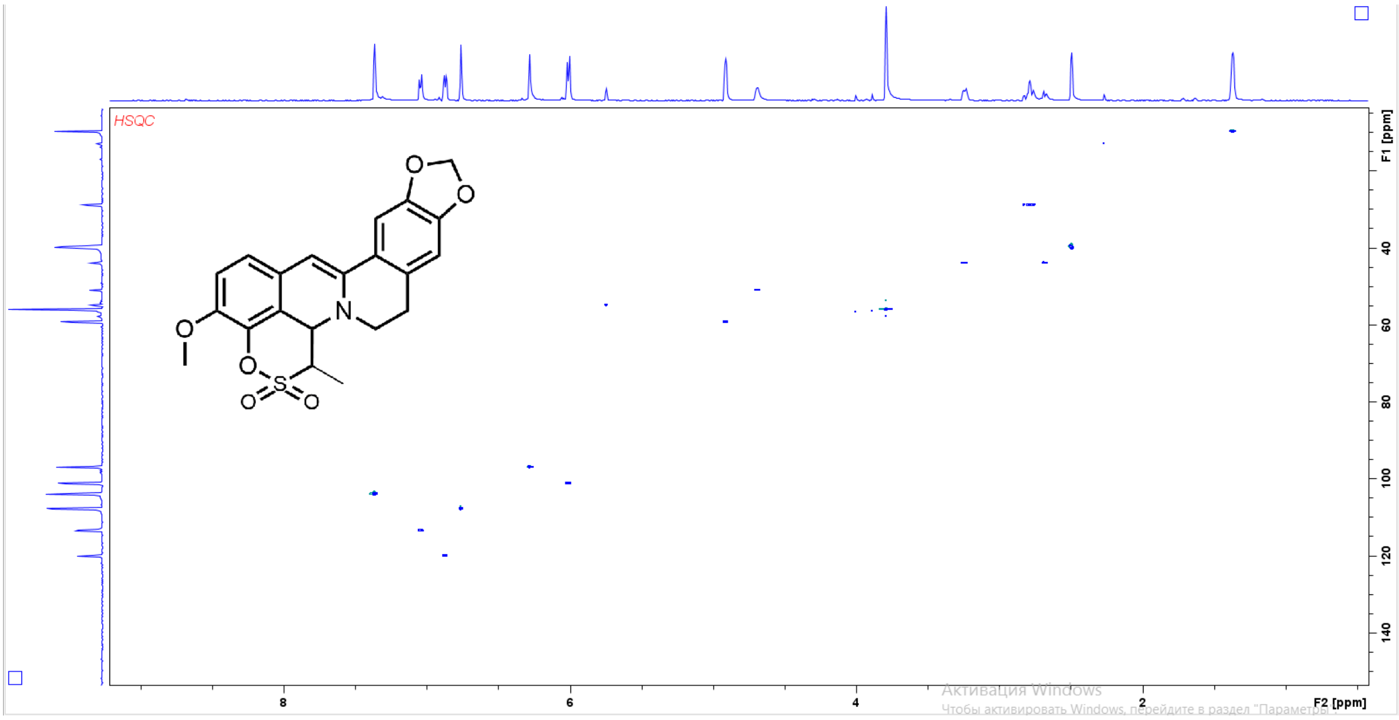

**Table S1.** The binding affinities as predicted by the scoring functions used.

| Molecules | **ASP** | **ChemScore** | **GoldScore** | **ChemPLP** | **$IC_{50}$, μM** |
|---|---|---|---|---|---|
| **5a** | 34.2 | 29.0 | 52.1 | 53.9 | 1.53 |
| **5b** | 35.0 | 26.7 | 50.5 | 59.0 | 2.13 |
| **5c** | 35.2 | 29.4 | 52.4 | 59.7 | 5.5 |
| **5d** | 34.0 | 27.7 | 50.5 | 60.0 | 1.12 |
| **14b** | 35.4 | 27.6 | 51.1 | 59.4 | 1.13 |
| **14a** | 34.5 | 29.0 | 51.7 | 54.6 | 0.925 |
| **12a** | 34.8 | 28.9 | 69.0 | 60.8 | 2.5 |
| **14c** | 34.5 | 27.8 | 48.9 | 56.5 | 0.56 |
| **14d** | 34.9 | 28.3 | 51.1 | 56.5 | 0.78 |
| **17a** | 33.6 | 27.8 | 56.9 | 62.8 | >15 |
| **17b** | 35.1 | 28.4 | 58.0 | 63.2 | >15 |

**Table S2.** The molecular descriptors and their corresponding Known Drug Indexes 2a and 2b ($KDI_{2a/2b}$).

| **Ligand** | **RB** | **MW** | **HD** | **HA** | **Log P** | **PSA** | **$KDI_{2A}$** | **$KDI_{2B}$** | **$IC_{50}$, μM** |
|---|---|---|---|---|---|---|---|---|---|
| **5a** | 1 | 399.4 | 0 | 7.75 | 2.0 | 78.7 | 5.36 | 0.49 | 1.53 |
| **5b** | 1 | 413.4 | 0 | 7.75 | 2.3 | 77.0 | 5.36 | 0.49 | 2.13 |
| **5d** | 3 | 441.5 | 0 | 7.75 | 3.1 | 75.4 | 5.48 | 0.56 | 1.12 |
| **14b** | 1 | 492.3 | 0 | 7.75 | 2.9 | 77.0 | 5.16 | 0.39 | 1.13 |
| **14a** | 1 | 478.3 | 0 | 7.75 | 2.6 | 78.6 | 5.21 | 0.41 | 0.925 |
| **12a** | 1 | 397.4 | 0 | 7.25 | 2.1 | 78.6 | 5.42 | 0.52 | 2.5 |
| **14c** | 3 | 520.4 | 0 | 7.75 | 3.6 | 75.3 | 5.19 | 0.39 | 0.56 |
| **$R^2$** | 0.3242 | 0.735 | | | 0.613 | 0.257 | 0.356 | 0.341 | |

**Table S3**. Definition of lead-like, drug-like and Known Drug Space (KDS) in terms of molecular descriptors. The values given are the maxima for each descriptor for the volumes of chemical space used.

| | **Lead-like Space** | **Drug-like Space** | **Known Drug Space** |
|---|---|---|---|
| Molecular weight (g $mol^{-1}$) | 300 | 500 | 800 |
| Lipophilicity (Log P) | 3 | 5 | 6.5 |
| Hydrogen bond donors (HD) | 3 | 5 | 7 |
| Hydrogen bond acceptors (HA) | 3 | 10 | 15 |
| Polar surface area ($Å^2$) (PSA) | 60 | 140 | 180 |
| Rotatable bonds (RB) | 3 | 10 | 17 |
